# Mucosal transcriptomics highlight lncRNAs implicated in ulcerative colitis, Crohn’s disease, and celiac disease

**DOI:** 10.1172/jci.insight.170181

**Published:** 2023-07-24

**Authors:** Tzipi Braun, Katya E. Sosnovski, Amnon Amir, Marina BenShoshan, Kelli L. VanDussen, Rebekah Karns, Nina Levhar, Haya Abbas-Egbariya, Rotem Hadar, Gilat Efroni, David Castel, Camila Avivi, Michael J. Rosen, Anne M. Grifiths, Thomas D. Walters, David R. Mack, Brendan M. Boyle, Syed Asad Ali, Sean R. Moore, Melanie Schirmer, Ramnik J. Xavier, Subra Kugathasan, Anil G. Jegga, Batya Weiss, Chen Mayer, Iris Barshack, Shomron Ben-Horin, Igor Ulitsky, Anthony Beucher, Jorge Ferrer, Jeffrey S. Hyams, Lee A. Denson, Yael Haberman

**Affiliations:** 1 Sheba Medical Center, Tel-Hashomer, affiliated with the Tel Aviv University, Tel Aviv, Israel.; 2 Faculty of Medicine, Tel Aviv University, Tel Aviv, Israel.; 3 Cincinnati Children’s Hospital Medical Center, Department of Pediatrics, University of Cincinnati College of Medicine, Cincinnati, Ohio, USA.; 4 Center for Pediatric IBD and Celiac Disease, Department of Pediatrics, Stanford University School of Medicine, Stanford, California, USA.; 5 Hospital for Sick Children, Toronto, Canada.; 6 Children’s Hospital of East Ontario, Ottawa, Ontario, Canada.; 7 Nationwide Children’s Hospital, Columbus, Ohio, USA.; 8 Department of Pediatrics and Child Health, Aga Khan University, Karachi, Pakistan.; 9 Department of Pediatrics, University of Virginia, Charlottesville, Virginia, USA.; 10 The Technical University of Munich, Munich, Germany.; 11 Broad Institute of MIT and Harvard University, Cambridge, Massachusetts, USA.; 12 Massachusetts General Hospital, Harvard Medical School, Boston, Massachusetts, USA.; 13 Emory University, Atlanta, Georgia, USA.; 14 Department of Computer Science, Cincinnati Children’s Hospital Medical Center and the University of Cincinnati College of Engineering, Cincinnati, Ohio, USA.; 15 Departments of Biological Regulation and Molecular Neuroscience, Weizmann Institute of Science, Rehovot, Israel.; 16 Section of Genetics and Genomics, Department of Metabolism, Digestion and Reproduction, Imperial College London, London, United Kingdom.; 17 Regulatory Genomics and Diabetes, Centre for Genomic Regulation, the Barcelona Institute of Science and Technology, Barcelona, Spain.; 18 Centro de Investigación Biomédica en red Diabetes y enfermedades metabólicas asociadas (CIBERDEM), Spain.; 19 Connecticut Children’s Medical Center, Hartford, Connecticut, USA.

**Keywords:** Gastroenterology, Inflammatory bowel disease

## Abstract

Ulcerative colitis (UC), Crohn’s disease (CD), and celiac disease are prevalent intestinal inflammatory disorders with nonsatisfactory therapeutic interventions. Analyzing patient data-driven cohorts can highlight disease pathways and new targets for interventions. Long noncoding RNAs (lncRNAs) are attractive candidates, since they are readily targetable by RNA therapeutics, show relative cell-specific expression, and play key cellular functions. Uniformly analyzing gut mucosal transcriptomics from 696 subjects, we have highlighted lncRNA expression along the gastrointestinal (GI) tract, demonstrating that, in control samples, lncRNAs have a more location-specific expression in comparison with protein-coding genes. We defined dysregulation of lncRNAs in treatment-naive UC, CD, and celiac diseases using independent test and validation cohorts. Using the Predicting Response to Standardized Pediatric Colitis Therapy (PROTECT) inception UC cohort, we defined and prioritized lncRNA linked with UC severity and prospective outcomes, and we highlighted lncRNAs linked with gut microbes previously implicated in mucosal homeostasis. *HNF1A-AS1* lncRNA was reduced in all 3 conditions and was further reduced in more severe UC form. Similarly, the reduction of *HNF1A-AS1* ortholog in mice gut epithelia showed higher sensitivity to dextran sodium sulfate–induced colitis, which was coupled with alteration in the gut microbial community. These analyses highlight prioritized dysregulated lncRNAs that can guide future preclinical studies for testing them as potential targets.

## Graphical abstract

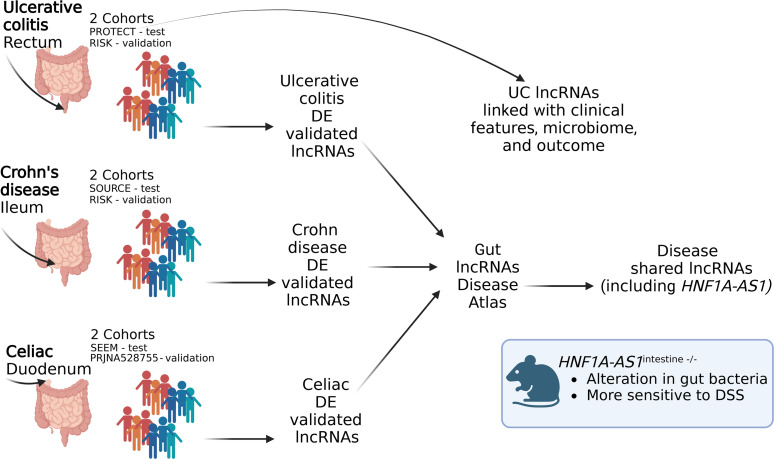

## Introduction

Ulcerative colitis (UC), Crohn’s disease (CD), and celiac disease are chronic inflammatory disorders affecting the intestine and are prevalent in Western countries (1–3). Patients with UC and CD require life-long therapy, but current treatments lead to sustained remission in fewer than 50% of cases (4, 5), while in celiac, patients require lifelong strict adherence to a gluten-free diet affecting their quality of life (6). In light of the increasing prevalence and disease burden, novel avenues of investigation are necessary to identify potential therapeutic targets to improve the treatment of patients with these conditions. lncRNAs have been implicated in human diseases (7–9) and were shown to play a key role in transcription regulation (10–13), metabolic pathways (9, 14, 15), and intestinal barrier functions (16, 17). The majority of lncRNAs exhibit expression patterns more tissue- and cell-specific than protein-coding genes (18), and they are readily targetable by RNA therapeutics. This, together with their regulatory role, makes them potentially attractive targets for intervention with limited adverse side effects.

We have used large and well-characterized cohorts of treatment-naive patients, free from confounding variables of previous therapy, to characterize transcriptomics mucosal signals linked with CD (19, 20), UC (21), and celiac disease (22, 23) focusing on the protein-coding genes. We now expand these analyses to identify lncRNAs whose expression was dysregulated in CD, celiac, and UC using independent test and validation cohorts (696 mucosal mRNA-Seq), which were reanalyzed uniformly starting from the raw data. mRNA-Seq used polyA selection and, therefore, captures most but not all lncRNAs, although nonpolyadenylated RNAs are generally highly unstable (24). We identified differentially expressed (DE) genes within cohorts and then cross-compared the results, generating a comprehensive atlas of dysregulated lncRNAs in treatment-naive UC, CD, and celiac disease. We highlight lncRNA expression along the gastrointestinal (GI) tract, demonstrating that, in control samples, lncRNAs have a more location-specific expression in comparison with protein-coding genes. Finally, using the Predicting Response to Standardized Pediatric Colitis Therapy (PROTECT) inception cohort study, we defined and prioritize lncRNAs linked with UC severity and prospective outcome, and tried to infer their function using their coexpressed better annotated protein-coding genes. Additionally, we highlighted lncRNAs that were associated with shifts in microbes previously implicated in mucosal homeostasis. Besides using these sets of lncRNAs as biomarkers for diagnosis, severity, and outcome, they also have the potential to be used as targets for interventions aimed to improve outcomes in patients who do not respond to available therapies.

## Results

### Identification of lncRNAs dysregulated in UC rectum, CD ileum, and celiac duodenum.

**Figure 1:**
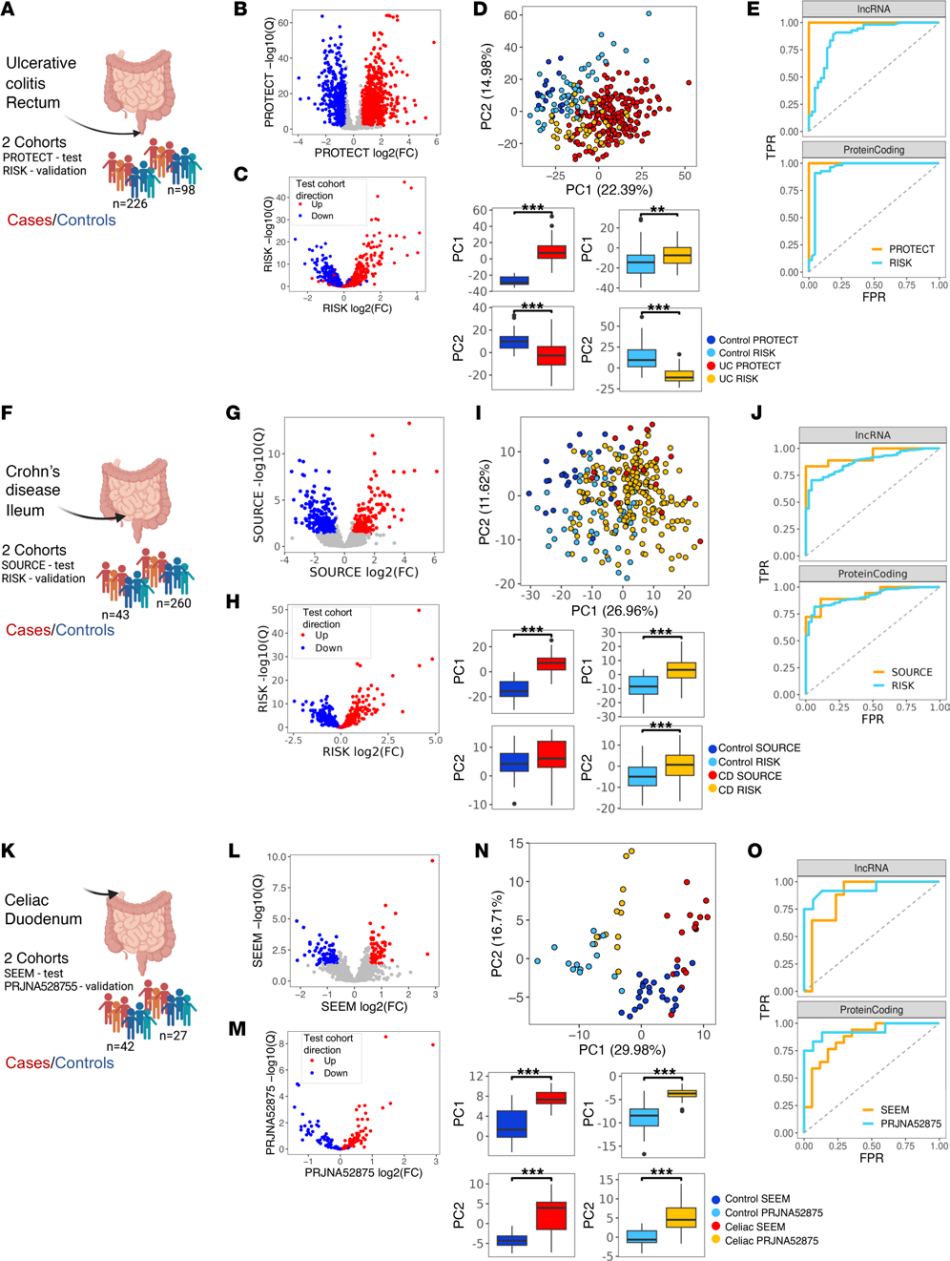
Dysregulated lncRNAs atlas in UC rectum, CD ileum, and celiac duodenum. (**A**, **F**, and **K**) Schemes of test and validation cohorts used for each disease (Supplemental Table 1). (**A**) UC rectum PROTECT test (206 UC and 20 controls) and RISK validation (43 UC and 55 controls). (**F**) CD ileum SOURCE test (18 CD and 25 controls) and RISK validation (213 CD and 47 controls). (**K**) Celiac duodenum SEEM test (17 celiac cases and 25 controls) and PRJNA528755 validation (12 celiac and 15 controls). (**B**, **G**, and **L**) Volcano plots of differentially expressed lncRNAs between disease and control in the test cohorts: PROTECT, SOURCE, SEEM (FC ≥ 1.5, FDR ≤ 0.05). (**C**, **H**, and **M**) Volcano-like plots of the validation cohorts, showing log (FC) and –log_10_(Q value) values for DE genes obtained in the test cohorts: RISK UC (**C**), RISK CD (**H**), PRJNA528755 celiac (**M**). The direction of change in the test cohort is marked by color. (**D**, **I**, and **N**) PCA of test and validation cohorts: PROTECT and RISK UC (**D**), SOURCE and RISK CD (**I**), SEEM and PRJNA528755 celiac (**N**). lncRNA that passed DE in the test cohorts were used. PC1 and PC2 values box plots for cases and controls within each cohort. (**E**, **J**, and **O**) Receiver operating characteristic (ROC) curves of random forest (RF) analysis trained on the test cohort and tested on both test and validation cohorts showing accurate classification of most cases and controls using either lncRNA or protein-coding gene expression: UC rectum (**E**), CD ileum (**J**), celiac duodenum (**O**). **P < 0.01, ***P < 0.001, Mann-Whitney *U* test.

We aimed to generate a list of lncRNAs and protein-coding genes that were dysregulated in UC rectum, CD ileum, and celiac duodenum, including several treatment naive cohorts with transcriptomics data sets. We used a unified analyses pipeline, including 696 mRNA mucosal biopsy samples in both main and validation cohorts (Supplemental Figure 1A and Supplemental Table 1; supplemental material available online with this article; https://doi.org/10.1172/jci.insight.170181DS1). In all cohorts, mucosal biopsies were obtained prior to medical therapy and underwent high-throughput mRNA-Seq (mRNA-Seq). Differential analyses between treatment-naive cases and controls were performed independently within each cohort and were based on the main test cohorts (Supplemental Table 1, Supplemental Figure 1): PROTECT UC (Supplemental Table 2), SOURCE CD (Supplemental Table 3), and SEEM celiac (Supplemental Table 4). The analyses identified 1,278 lncRNAs DE between UC (*n* = 206) and control (Ctl) (*n* = 20) in PROTECT (Figure 1, A–E), 287 lncRNAs DE between CD (*n* = 18) and Ctl (*n* = 25) in SOURCE (Study Of Urban and Rural Crohn Disease Evolution) (Figure 1, F–J), and 146 lncRNAs DE between celiac (*n* = 17) and Ctl (*n* = 25) in SEEM (Study of Environmental Enteropathy and Malnutrition) (Figure 1, K–O). Verification of those main test cohorts DE lncRNAs in the corresponding validation cohorts— RISK cohort with 213 CD and 47 ileal Ctls and the 43 UC 55 rectal Ctls, and PRJNA528755 (ref. 25) with 12 celiac and 15 duodenal Ctls — shows an overall good consistency. Volcano plots of the validation cohorts, using lncRNAs that passed DE in the main cohorts, showed a direction of change consistent with that seen in the main cohort (red indicates increased and blue indicates decreased in disease lncRNAs; Figure 1, B, C, G, H, L, and M), and scatter plots of the log_2_(fold change [FC]) values calculated between cases and Ctl samples, for lncRNAs that passed DE in the main cohorts showed high correlation between test and validation cohorts (Supplemental Figure 1B). PCA for lncRNAs that passed DE in the main cohorts showed clustering of cases and controls in both the test and validation cohorts (Figure 1, D, I, and N), with significant separation of cases and controls across all 6 cohorts (test and validation) on both PC1 and PC2. Random forest (RF) analysis using the DE genes (identified in the main test cohorts) was trained on the test cohorts. The resulting model was then applied to the validation cohorts, with receiver operating characteristic (ROC) curves showing accurate classification of most cases and controls using either lncRNA or protein-coding gene expression (Figure 1, E, J, and O).

**Figure 2:**
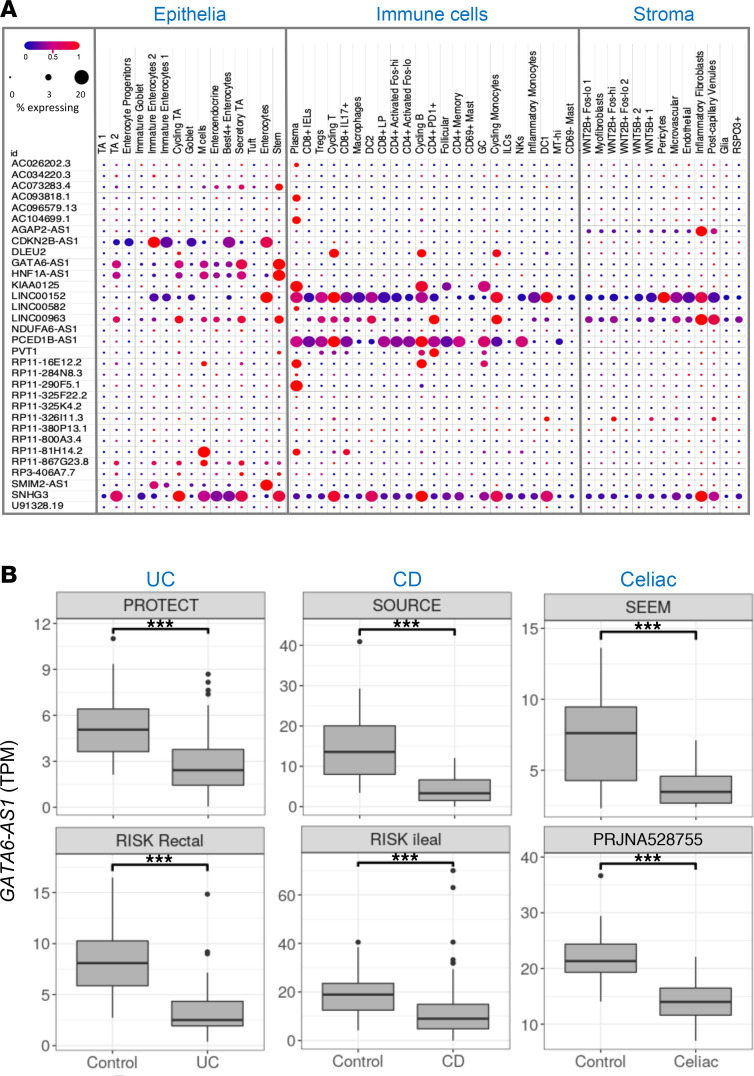
Expression of the lncRNAs in UC, CD, and celiac. (**A**) Cellular expression of the lncRNAs that were dysregulated in UC, CD, and celiac and included (32 of 45) in adult colon single-cell RNA-Seq data set (26). The size and color of the dots are proportional to the percentage of cells expressing the gene and the normalized expression, respectively. (**B**) Box plots showing *GATA6-AS1* expression between cases and control in all 6 cohorts: PROTECT UC (206 UC and 20 controls) and RISK UC rectal (43 UC and 55 controls), SOURCE (18 CD and 25 controls) and RISK CD ileal (213 CD and 47 controls), SEEM celiac (17 celiac cases and 25 controls), and the celiac cohort (PRJNA528755, 12 celiac and 15 controls). Similar box plots are available for all lncRNAs expressed in 1 of 3 main cohorts in https://tzipi.shinyapps.io/lncRNA_gut/ (28). ***P < 0.001, Mann-Whitney *U* test.

We next created a list of validated dysregulated lncRNAs and protein-coding genes. For within-disease validation and between-disease comparisons, lncRNAs and protein-coding genes were considered validated if they showed a similar direction of change and passed the predefined fold change and FDR cutoffs in both test and validation cohorts (Supplemental Figure 1A and Supplemental Data 1). Based on these criteria, 451 lncRNAs were validated in UC rectum, 276 lncRNA were validated in CD ileum, and 42 lncRNA were validated in celiac duodenum. Furthermore, based on these criteria, we identified 45 lncRNAs and 911 protein-coding genes that showed similar dysregulation in all three diseases: UC, CD, and celiac. To examine cell-specific trends in gene expression of these lncRNAs, we analyzed a publicly available adult human colon UC single-cell data set (26) (Figure 2A) and a pediatric CD single-cell data set (27) (Supplemental Figure 2). We observed cell-specific expression of several lncRNAs including *GATA6-AS1*, *SMIM2-AS1*, and *CDKN2B-AS1* in gut epithelia, *RP11 290F5.1* and *KIAA0125* to plasma cells and B cells, and LUCAT1 and LINC01272 to Myeloid linage. A subgroup of lncRNAs was also further validated using quantitative PCR (qPCR) in biopsies or rectal organoids versus peripheral leukocytes (peripheral blood mononuclear cells [PBMCs]) of Ctl subjects (Supplemental Figure 2, B and C). We generated an interactive lncRNAs platform (28) (https://tzipi.shinyapps.io/lncRNA_gut/) that can support further exploration of lncRNA expression and functions in these diseases; an example for the output using this platform is shown for *GATA6-AS1* (Figure 2B).

### lncRNAs show a more location-specific expression along the small and large intestine than protein-coding genes.

**Figure 3:**
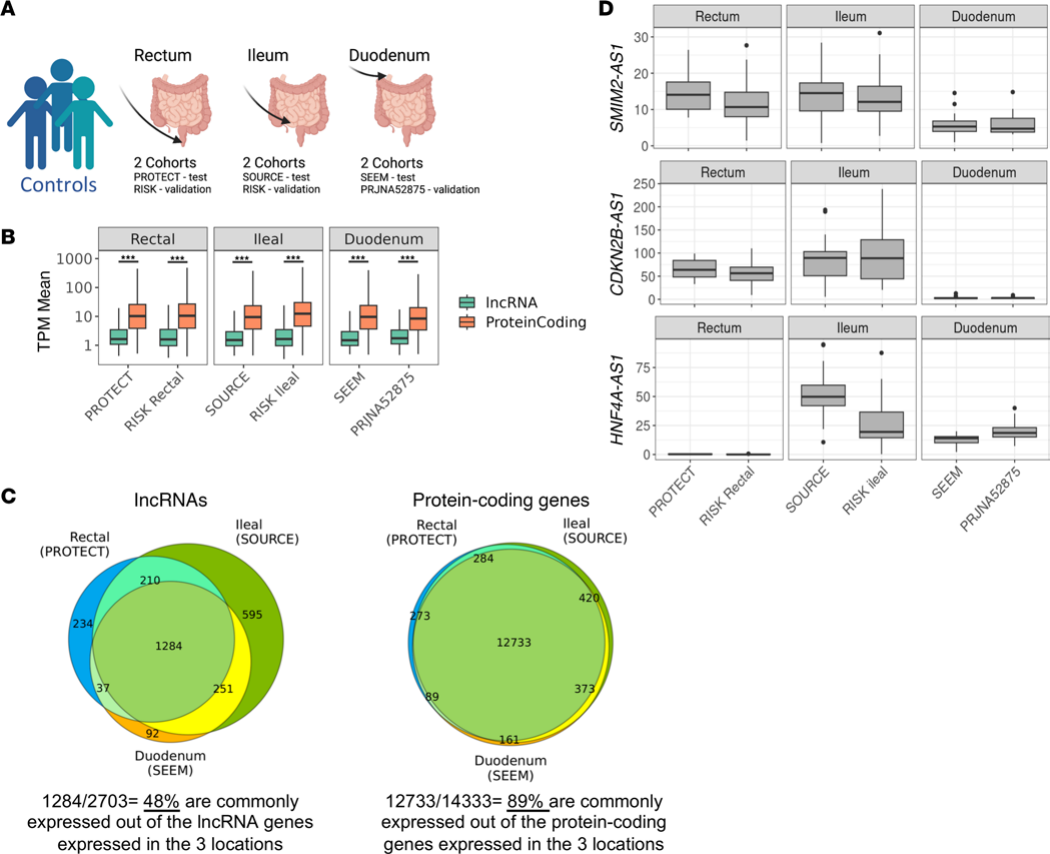
lncRNAs show more location-specific expression along the small and large intestine than do protein-coding genes. (**A**) Scheme of the control samples from 3 main and 3 validation cohorts: 2 rectum cohorts (PROTECT, *n* = 20, RISK, *n* = 55), 2 ileum cohorts (SOURCE, *n* = 25, RISK, *n* = 47), and 2 duodenum cohorts (SEEM, *n* = 25, PRJNA52875, *n* = 15). (**B**) Box plot showing the mean TPM values of all expressed protein-coding genes and lncRNAs of these control samples. ***q < 0.001, Mann-Whitney *U* test with FDR correction. (**C**) Venn diagrams indicating the number of expressed lncRNAs (left) and protein-coding genes (right) in control samples in the 3 main test cohorts that were processed similarly (TPM > 1 in at least 20% of samples); 48% of all lncRNAs and 89% of protein-coding genes are shared along these 3 locations in the GI tract. (**D**) Examples of lncRNAs TPM values in controls using all 6 main test and validation cohorts, showing expression in control rectum, ileum, and duodenum (28). Graphs’ central lines indicate median and lateral lines represent upper and lower quartiles.

lncRNAs were previously shown to have a more tissue-specific expression in comparison with protein-coding genes (18). We, therefore, tested the location-specific expression along the GI tract, including the proximal (duodenum) and distal (ileum) small intestine, and the large intestine (colon), of the lncRNAs and protein-coding genes. To avoid a confounding effect of disease, we used only mucosal biopsies from controls (Supplemental Figure 3, A and B). The median TPM expression of lncRNAs was significantly lower (Mann-Whitney *U* test *P* < 0.001) than that of protein-coding genes in all locations and cohorts, as expected (29) (Figure 3, A and B). Direct comparison of the expressed lncRNAs and protein-coding genes along these locations, using only the test cohorts that were processed similarly (similar methodology and library preparation in the same core facility Supplemental Table 1, and Supplemental Figure 3), revealed that the lncRNAs showed significantly more location-specific expression along the GI tract than did the protein-coding genes (Figure 3C and Supplemental Data 2; 1,269 of 2,650 [48%] lncRNAs compared with 12,698 of 14,249 [89%] of protein-coding genes, were expressed in all 3 intestinal locations, χ^2^ test, *P* < 0.001). To confirm that this is not just a detection issue due to the inherent lower expression of lncRNAs, we checked expression percentage at a lower TPM cutoff of 0.1 (compared with our usual 1 TPM in 20% of samples) showing that the lncRNAs included in our analysis using the lower cutoff were expressed in over 75% of samples, while the discarded lncRNAs did not seem to have a higher expression than protein-coding genes. Additionally, there was a good correlation of lncRNA expression between the main and validation cohorts (Supplemental Figure 3, C–E). Examples of lncRNA expression in controls using these data sets are shown (Figure 3D) and can be extracted also from the interactive lncRNAs platform (28).

### lncRNAs show a strong association with UC severity and outcome.

**Figure 4:**
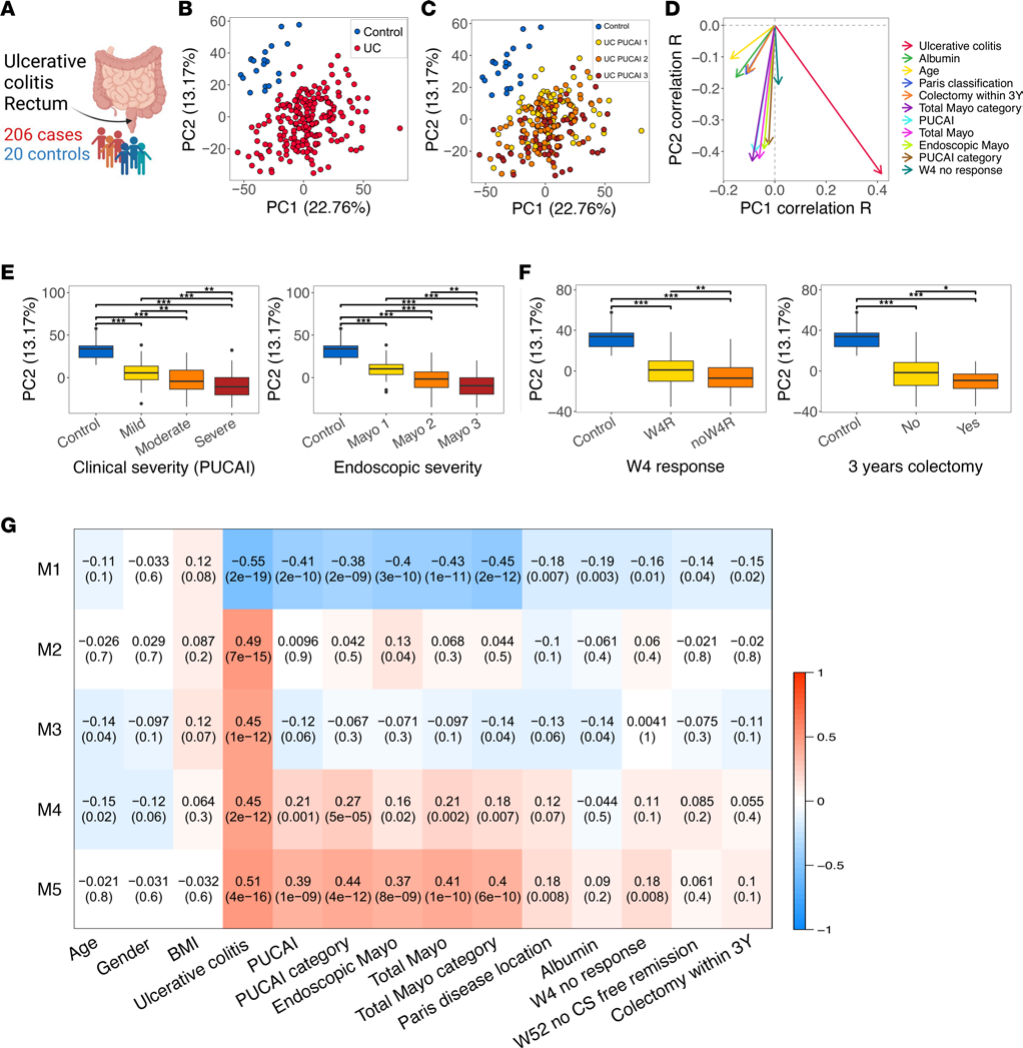
Ulcerative colitis mucosal transcriptomes reveal lncRNA landscape linked with personalized disease severity and treatment response. (**A**) Scheme for the PROTECT transcriptomics (206 UC and 20 controls). The scheme was created with biorender.com. (**B** and**C**) PCA using 2,378 lncRNAs that passed expression filtering in PROTECT, colored by diagnosis (**B**) and disease severity (**C**). Pediatric Ulcerative Colitis Activity Index (PUCAI): mild, 10−30; moderate, 35−60; and severe, 65 or higher. (**D**) Spearman’s correlation between clinical metadata and lncRNAs PCA’s PC1 (22.8% variation) and PC2 values (13.2% variation) showing significant correlations with PC1 or PC2 values (*P* ≤ 0.05); PC1 and PC2 *r* values mark the arrowhead *x* axis and *y* axis coordinates, respectively. (**E**) Box plots of PC2 values stratified by clinical (left, PUCAI; 53 mild, 85 moderate, 68 severe UC cases) and endoscopic severity (right, endoscopic Mayo score: 27 Mayo 1, 108 Mayo 2, 71 Mayo 3 UC cases stratified by endoscopic severity). (**F**) Box plots of PC2 values stratified by UC course (left, week 4 remissions after 5-ASA/steroids: 105 W4R and 101 no W4R; right, colectomy within 3 years: 189 no colectomy and 17 had colectomy). *q < 0.05, **q < 0.01 ***q < 0.001, calculated using Mann-Whitney *U* test with Benjamini-Hochberg FDR correction. (**G**) WGCNA lncRNAs coexpression modules heatmap (represented by module eigengenes and numbered M1–M5), which were correlated with UC diagnosis (*P* < 0.001, Supplemental Data 1 includes all modules) and the indicative clinical features. Data are shown as the correlation coefficient and *P* value for each comparison. All clinical data besides the outcome data are from the time of diagnosis. Graphs’ central lines indicates median and lateral lines represent upper and lower quartiles.

The PROTECT UC inception cohort was designed to examine factors associated with baseline disease severity and with responses to standardized therapy and to highlight mucosal signals and genes that are linked with nonresponse to therapy. In PROTECT, UC was clinically and endoscopically graded at baseline, and disease course was recorded prospectively after standardized initial therapy with mesalamine or corticosteroids (21, 30, 31) (Figure 4A and Supplemental Table 2). Focusing on the protein-coding genes in PROTECT, we previously reported a profound suppression of mitochondrial genes and function across cohorts in active UC (21), and we defined genes and pathways linked to UC early and late course (21, 30, 31). PROTECT transcriptomics included 2,378 annotated lncRNAs. Using these 2378 lncRNAs in a PCA plot helped differentiate between UC and controls on both PC1/PC2 (Figure 4, B–D). Additionally, PCA PC2 showed a significant correlation (Figure 4D) with clinical disease severity (Pediatric Ulcerative Colitis Activity Index [PUCAI]), endoscopic disease severity (endoscopic Mayo score), and early (week 4 remission after 5-ASA/steroids [W4R]) and late disease course (colectomy within 3 years). Significant lower PCA PC2 values were noted in patients with more severe disease (Figure 4E) and less favorable outcomes (Figure 4F).

Using the weighted gene coexpression network analysis (WGCNA) (32), we explored the relationships (22) between lncRNA gene coexpression modules and UC severity, albumin level (positively linked with better outcome; ref. 33), and disease course including early response to therapy (W4R), week 52 steroid-free remission (W52SFR), and colectomy within 3 years. This systems biology approach identifies clusters (modules) of highly correlated genes that, when linked to sample traits, such as disease severity and outcome, can highlight candidate biomarkers and therapeutic targets. WGCNA was applied using only the lncRNA (numbered modules) to prioritize and explore those linked to UC and disease severity, independently of protein-coding genes. This resulted in 5 lncRNA modules (M1–M5) associated with UC (*P* < 0.001; Figure 4G, Supplemental Figure 4, and Supplemental Data 3). M1 and M4–M5 showed significant associations with clinical (PUCAI) and endoscopic severity (Mayo score). M1, which contained lncRNAs reduced in UC (i.e., *GATA6-AS1*, *CDKN2B-AS1*, and *HNF1A-AS1* expressed in epithelial cells) was significantly associated with poorer course — specifically, with no remission at W4 (*P* = 0.01), no W52SFR (*P* = 0.04), and a need for colectomy (*P* = 0.02). By contrast, M2–M5 contained lncRNAs that were increased in UC, but only M5 — which includes *LINC01272 —* showed a significant association with poorer outcome of no W4R (*P* = 0.008). Using different combinations of IFN-γ and IL-1b that are highly upregulated in UC and LPS, which is a bacterial product increased in the circulation of patients with UC (34), resulted in the induction of *DUOX2* and *CXCL8* (IL-8) in HT-29 colonic-derived cell line (Supplemental Figure 4), and we were able to confirm reduction and induction of specific lncRNAs from M1, M2, and M5 using this models system.

**Figure 5:**
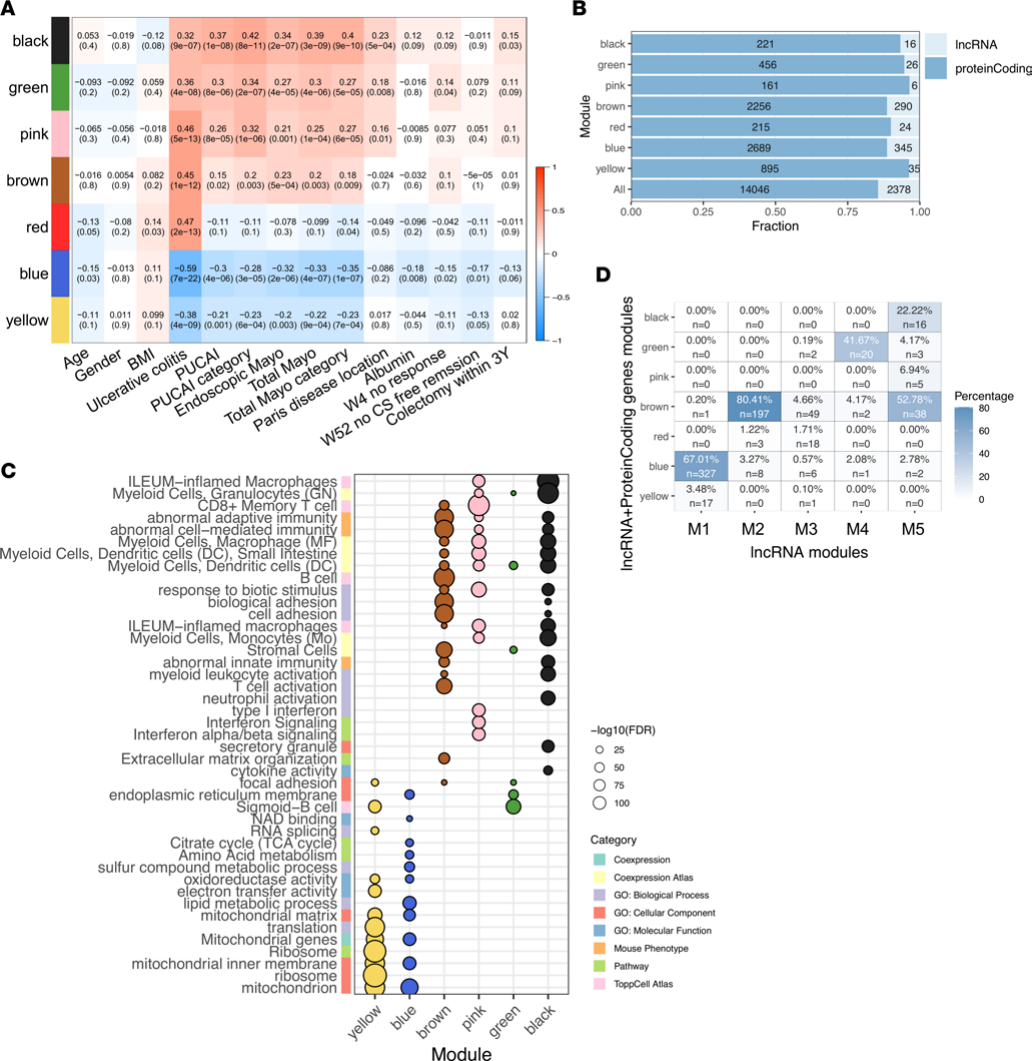
lncRNA prioritization and inferred function from coexpression with protein-coding genes. (**A**) WGCNA coexpression modules heatmap that includes lncRNAs and protein-coding genes, generated using the PROTECT cohort (206 UC and 20 controls). Modules that were correlated with UC diagnosis (*P* < 0.001) and other clinical features are shown. Numbers represent the correlation coefficient and *P* value for each comparison. (**B**) For each WGCNA module associated with disease and for all modules combined, the fraction of lncRNAs and protein-coding genes are marked on the *x* axis, and the actual number of genes is written within the bar. (**C**) ToppGene/ToppCluster functional annotation enrichment of protein-coding genes within each module. FDR is shown as circle size; selected annotations origin database is marked on the *y* axis (full list in Supplemental Data 1). (**D**) Heatmap showing the overlap between UC lncRNA–only modules (numbered M1–M5), and UC lncRNA plus protein-coding gene modules (colored). For each lncRNA module, the number of lncRNAs shared between lncRNAs plus protein-coding gene modules is noted as well as the percentage of those lncRNAs of the lncRNAs-only WGCNA modules.

To infer potential lncRNA functions from their coexpressed and better annotated protein-coding genes, we used WGCNA and integrated both the 14,046 protein-coding and 2,378 lncRNA genes, applying an additional step of functional annotation enrichment analysis within the expression modules. This step is performed to infer the functionality of the modules, using protein-coding genes that are better annotated than lncRNA. This resulted in 7 modules associated with UC (*P* < 0.001; Figure 5, A and B, and Supplemental Data 3), with no apparent enrichment of lncRNAs in a particular module (Figure 5B). Six of the 7 modules showed significant association with clinical and endoscopic severity, but only the blue downregulated module showed significant associations with overall poorer outcome (W4R and W52SFR), whereas the black and green upregulated modules showed association with colectomy and W4R, respectively (*P* < 0.05). Functional annotation enrichment analysis of the various modules (Figure 5C, Supplemental Figure 5, and Supplemental Data 3) revealed enrichment for mitochondrial genes in both blue and yellow downregulated UC modules, with more specific enrichment for TCA cycle and lipid and amino acid metabolism in the blue module. The black, green, brown, and pink upregulated modules showed some overlaps in enrichment for innate and adaptive immune response to biotic stimuli, whereas the black module showed the strongest enrichment to myeloid innate response, the green to B cells, the brown to stromal cells and adhesion, and the pink to type 1 interferon signaling. The intersection of the modules in Figure 4G (only lncRNAs) and Figure 5A (both lncRNAs and protein-coding) to prioritize the lncRNA more likely to be functional (Figure 5D and Supplemental Data 3) indicated that 327 lncRNAs from M1 were included in the blue module enriched for mitochondrial TCA and lipid metabolism, and another ± 300 lncRNAs were included in M2, M4, and M5 modules that are linked with immune responses. These results are in agreement with the qPCR validation (Supplemental Figure 2, B and C), where *HNF1A-AS1* lncRNA from M1 module, which was associated with the blue epithelial module, was specifically more enriched in rectum-derived epithelial organoids than in the PBMCs (Supplemental Figure 2C), whereas LUCAT1 lncRNA from M5 module — which associated with black, green, and brown modules that were linked to immune responses — was enriched in the PBMCs (Supplemental Figure 2B). Similarly, *HNF1A-AS1* was reduced and *LUCAT1* was induced upon inflammatory triggers (Supplemental Figure 4B).

**Figure 6:**
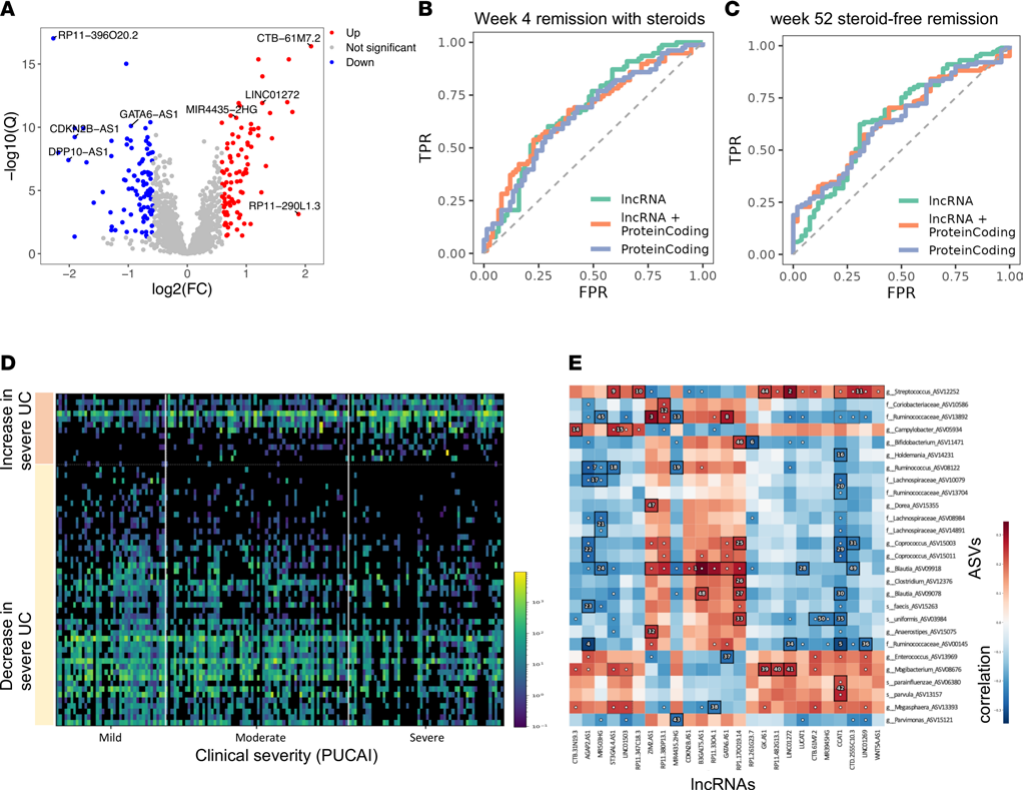
lncRNAs showing UC severity predict outcomes similar to protein-coding genes and are associated with the gut microbiome. (**A**) Volcano plot of the 192 differentially expressed lncRNAs between 53 patients with mild UC and 68 patients with severe UC (FC ≥ 1.5, FDR ≤ 0.05). (**B** and **C**) Receiver operating characteristic (ROC) curves of random forest (RF) analysis using either the 960 protein-coding genes or the 192 lncRNA severity-associated genes, or both lncRNAs and protein-coding genes, for W4 early response and for W52SFR in the moderate-severe patients’ group (*n* = 153) that received standardized initial treatment with corticosteroids. The graph showed 1 representative iteration out of 100 RF performed iterations. The mean ROC AUC for W4, using the lncRNA was 0.68 (min, 0.65; max, 0.70), which was similar to those obtained using only the protein-coding genes (mean, 0.67; min, 0.65; max, 0.69), and those obtained using both lncRNAs and protein-coding genes (mean, 0.67; min, 0.65; max, 0.69). For W52SFR, the mean ROC AUC using the lncRNA was 0.63 (min, 0.60; max, 0.66), the mean ROC AUC using the protein-coding was 0.65 (min, 0.63; max, 0.67) and the mean ROC AUC using both lncRNAs and protein-coding genes was 0.65 (min, 0.62; max, 0.67). (**D**) Heatmap showing significant differential bacterial ASVs (47 ASVs more abundant in mild and 12 more abundant in severe cases) between 38 samples from patients with mild UC and 54 samples from patients with severe UC (rank-mean test with FDR < 0.1). Each row represents an ASV, and each column is a patient sample (38 mild, 64 moderate, 54 severe). (**E**) Heatmap summarizing the association between lncRNA expression and microbial ASV abundance using HAllA testing, with FDR < 0.1, using the 156 samples with matching microbial ASV and lncRNA expression data.

More severe disease has been linked with higher rates of therapy escalation and colectomy (35, 36). To capture more directly lncRNAs linked with clinical disease severity, we identified 192 lncRNAs and 960 protein-coding genes that differed significantly between severe and mild cases at diagnosis (FDR ≤ 0.05 and FC ≥ 1.5; Figure 6A and Supplemental Data 4). For insight regarding cell-specific trends in lncRNA expression, we analyzed a publicly available adult human colon UC single-cell data set (26), which indicated that the lncRNAs reduced in severe versus mild cases showed predominant epithelial expression (Supplemental Figure 6), while lncRNAs induced in severe patients showed a more variable expression in epithelia, stroma, and immune cells. Importantly, using the PROTECT cohort, we have already shown no difference in epithelial cells abundance, but a profound suppression of specific genes and protein like mitochondrial proteins in epithelial cells, largely independent of clinical severity in active UC (21). Clinical factors associated with early and late UC disease course included baseline disease severity and initial therapy (31). We therefore focused our W4 and W52SFR outcome analysis on the moderate-severe patients who received initial corticosteroids treatment. Using RF analysis to estimate outcome classification, we compared the ROC curve using either the severity associated 960 protein-coding genes, 192 lncRNAs, or both (Figure 6, B and C). After applying 100 different RF iterations (Supplemental Data 4 highlight genes contributing to classification) the mean area under the ROC curve (AUC) for W4, using lncRNAs, was 0.68 (minimum [min], 0.65; maximum [max], 0.70), which was comparable with using only protein-coding genes (mean, 0.67; min, 0.65; max, 0.69) and with using both lncRNAs and protein-coding genes (mean, 0.67; min, 0.65; max, 0.69). For W52SFR, the mean AUC using lncRNAs was 0.63 (min, 0.60; max, 0.66), the mean AUC using protein-coding genes was 0.65 (min, 0.63; max, 0.67), and for both the mean was 0.65 (min, 0.62; max, 0.67). It appears that lncRNAs are comparable with protein-coding genes, as there was no significant difference between the protein-coding genes and lncRNAs in the AUC values. To further account for disease severity, we performed multivariable regression analyses for WK4 and W52 outcomes, including the clinical or endoscopic severity alone or together with PCA PC1 values that summarized variation in the 192 lncRNAs and 960 protein-coding genes (Figure 6A and Supplemental Data 4). Models that included the respective 192 lncRNAs and/or 960 protein-coding gene transcriptomics PC1 signal improved the AUC, indicating superiority to the model which included clinical severity alone. Overall, we highlight the role of lncRNAs in pathogenesis and in relation to disease severity. Since many lncRNAs function at the RNA levels and have a regulatory role, this may imply the possibility of targeting them as new potential therapies.

Aberrant immune responses and shifts in gut microbes likely play a role in UC and treatment response. We previously showed that protein-coding genes linked with severity and therapeutic response gene signatures were associated with shifts in microbes previously implicated in mucosal homeostasis. We now tested which of the severity-associated lncRNAs are also correlated with the microbial composition. In total, 156 of the 206 patients with UC in our cohort also had fecal microbial profiles (37). Reanalyzing this data set resulted in 59 bacterial amplicon sequence variants (ASVs) significantly different (FDR < 0.1) between severe versus mild UC disease (Figure 6D and Supplemental Data 4). Microbial data were available only for the UC subgroup. We used hierarchical all-against-all association testing (HAllA) to identify specific significant associations between lncRNAs or protein-coding genes, and these microbial ASVs (FDR < 0.1), including positive associations between *Blautia* (ASV09078) and lncRNAs reduced in UC, including *GATA6-AS1*, and between Streptococcus and lncRNAs increased in UC, including *LINC01272* (Figure 6E, Supplemental Figure 7, and Supplemental Data 4).

### HNF1A-AS1 reduction in human and mice are linked with more severe UC forms and higher sensitivity to DSS-induced colitis, respectively.

**Figure 7:**
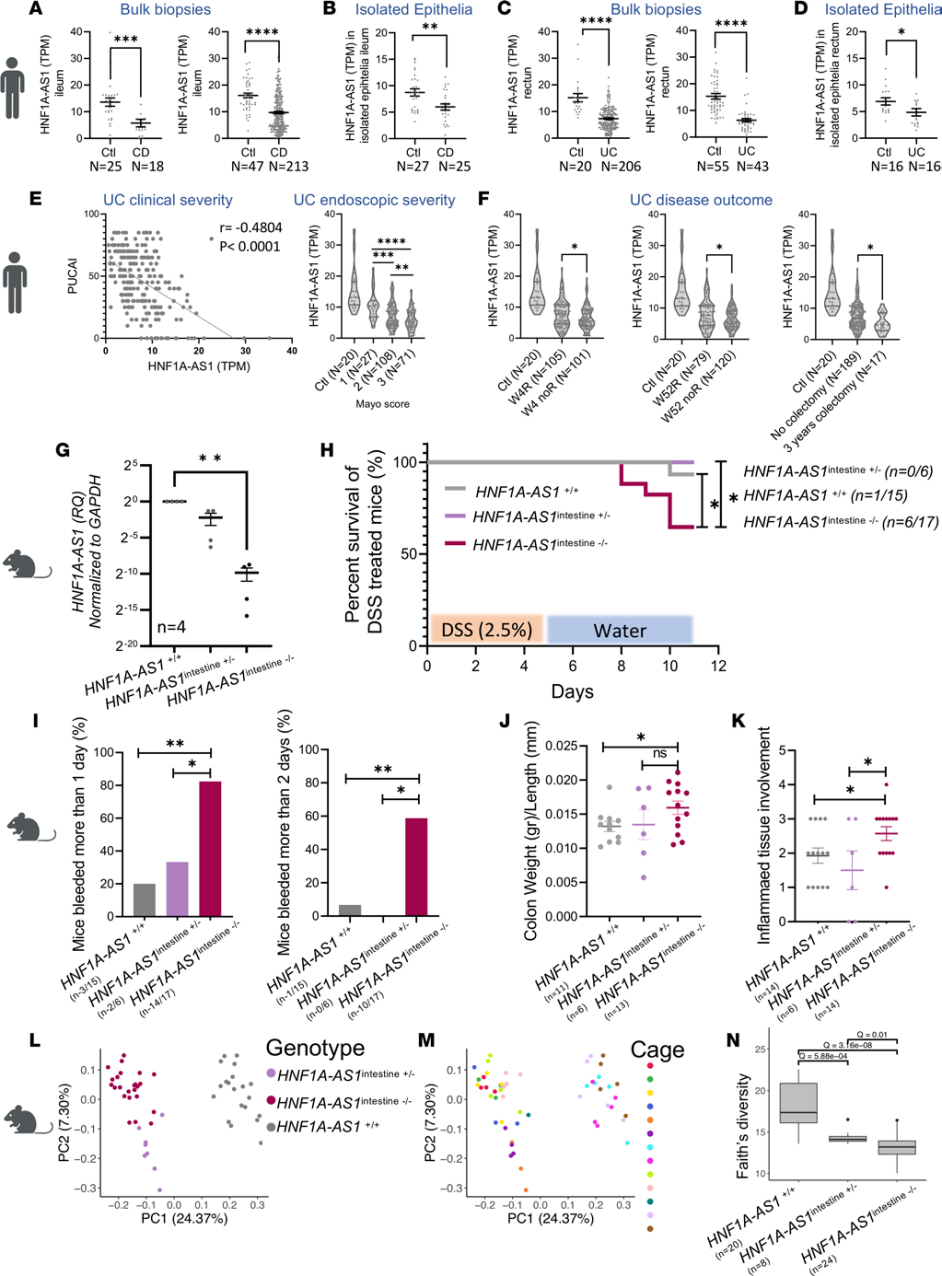
*HNF1A-AS1* reduction is linked with UC severity. (**A** and **B**) *HNF1A-AS1* expression is reduced in CD ileum (**A** and **B**) — SOURCE (18 CD, 25 control [Ctl]) and RISK (213 CD, 47 Ctl) bulk biopsies and isolated epithelia (38) (25 CD, 27 Ctl) — and in UC rectum (**C** and **D**) —PROTECT (206 UC, 20 Ctl) and RISK (43 UC, 55 Ctl) bulk biopsies and isolated epithelia (16 UC and 16 Ctl). (**E** and **F**) *HNF1A-AS1* at baseline is further reduced in UC cases with more severe clinical and endoscopic phenotype (**E**) and in those with less favorable outcome — week4 and week52 nonresponders (noR), or required colectomy within 3 years (**F**). Mice experiments included *HNF1A-AS1*^intestine–/–^ (intestine-specific deletion of the *HNF1A-AS1* promotor), *HNF1A-AS1*^+/+^, and *HNF1A-AS1*^intestine+/–^. (**G**) *HNF1A-AS1* was significantly reduced in rectal tissue of *HNF1A-AS1*^intestine–/–^ in comparison *HNF1A-AS1*^+/+^, Kruskal-Wallis test with Dunn’s correction, *n* = 4. Mice were treated with DSS (2.5%) for 5 days, followed by 6 days of water washout. (**H**) Kaplan-Meier survival curve during the experiment and differences between groups were calculated using the Mantel-Cox test. (**I**) Rectal bleeding was recorded (Left: bleeding duration more than one day. Right, bleeding duration more than 2 days). Differences were calculated using 2-sided Fisher exact test. (**J**) Colon weight to length (colon mass) at the end of the experiment. Histopathological evaluation using a predefined histologic scoring focusing on Inflammation score & percent of the involved region. (**K**) Differences between groups were tested using a 2-tailed *t* test. (**L** and **M**) PCoA Plot of fecal microbiome prior to the DSS treatment (Day 1) colored by mice group (**L**) or cage (**M**) *n* = 52. α Divesity (Faith’s phylogenetic) between *HNF1A-AS1*^+/+^ (*n* = 20), *HNF1A-AS1*^intestine+/–^ (*n* = 8), and *HNF1A-AS1*^intestine^^–/–^ (*n* = 24), prior to the DSS treatment (Day1). The *q* values were calculated using Mann-Whitney *U* test with FDR correction (**N**). *P ≤ 0.05, **P ≤ 0.01, ***P ≤ 0.001, ****P ≤ 0.0001 Mann-Whitney test. Graphs central line indicates median and lateral lines represent upper and lower quartiles.

*HNF1A-AS1* was within the 45 lncRNAs that showed similar dysregulation in all 3 diseases (Figure 2): UC, CD, and celiac. Moreover, *HNF1A-AS1* was included in the WGCNA M1 module, with relatively high MM scores (M1 = 0.882, eighth out of 488), and in the blue WGCNA module enriched for epithelial metabolic functions. Moreover, both the M1 (only lncRNAs) and the blue (includes both lncRNAs and protein-coding genes) modules were significantly related to UC outcome (Figure 4G and Figure 5A). We confirmed the reduction of *HNF1A-AS1* in human cohorts’ bulk biopsies and in an independent cohort that used isolated epithelial cells from biopsies of patients with CD and UC (38) (Figure 7, A–D). Moreover, we confirmed that further reduction of *HNF1A-AS1* is linked with more severe forms of UC (clinically and endoscopically; Figure 7E) and with less favorable outcomes, including less remission in week 4 and week 52 and more colectomy within 3 years from diagnosis (Figure 7F).

To explore and validate that the reduction of *HNF1A-AS1* is associated with more severe colitis forms and outcome and to test for a causal relationship between the reduction of *HNF1A-AS1* in gut epithelia and this phenotype, we took advantage of the fact that *HNF1A-AS1* is conserved and expressed in mouse intestine. We introduced an intestinal-specific deletion of *HNF1A-AS1* ortholog promotor in the C57BL/6J mice model, by crossing C57BL/6J *HNF1A-AS1*^loxP/–^ mice with VillinCre (39). This resulted in a robust reduction of *HNF1A-AS1* in intestinal tissue (*HNF1A-AS1*^intestine^^–/–^; Figure 7G) in comparison with Ctl mice (*HNF1A-AS1* ^+/+^). *HNF1A-AS1*^intestine^^+/–^, was used as a second control, where only 1 allele has the intestinal-specific deletion. Colitis was induced in *HNF1A-AS1* ^+/+^, *HNF1A-AS1*^intestine^^+/–^, and *HNF1A-AS1*^intestine^^–/–^mice by Dextran Sodium Sulphate–induced (DSS-induced) colitis that was added to water at a concentration of 2.5% (w/v) for 5 days, followed by 6 days of washout. Two independent experiments performed more than 2 months apart are jointly presented. *HNF1A-AS1*^intestine^^–/–^ mice showed more susceptibility to DSS colitis in comparison with *HNF1A-AS1* ^+/+^ and *HNF1A-AS1*^intestine^^+/–^, demonstrating significantly reduced survival (Figure 7H), more severe rectal bleeding (Figure 7I), higher colon weight/length ratio related to induced inflammation (Figure 7J), and larger extent of inflamed tissue as was scored in a blinded histopathologic evaluation of the H&E staining of the colon sections (Figure 7K and Supplemental Figure 8A).

Fecal content from these mice was evaluated for microbial composition. We analyzed the microbial community using 16S rRNA amplicon sequencing obtained from *HNF1A- AS1*^intestine^^–/–^, *HNF1A-AS1*^+/+^, and *HNF1A-AS1*^intestine^^+/–^. Samples were collected at several time points: on day 1 before DSS administration, and on day 6 and day 10 throughout the experiment. Principal coordinate analysis (PCoA) plot of day 1 fecal material prior to DSS treatment is shown in Figure 7L demonstrating clustering by genotype (*HNF1A-AS1*^+/+^, *HNF1A-AS1*^intestine^^+/–^, and *HNF1A-AS1*^intestine^^–/–^; Figure 7L). Each genotype cluster included samples from 2 different experiments (Supplemental Figure 8C), several cages (6 total cages for *HNF1A-AS1*^intestine^^–/–^, 3 for *HNF1A-AS1*^intestine^^+/–^, and 4 for *HNF1A-AS1*^+/+^; Figure 7M), several mothers (6 total mothers for *HNF1A-AS1*^intestine^^–/–^ and 5 for *HNF1A-AS1*^intestine^^+/–^), and litters (7 total litters for *HNF1A-AS1*^intestine^^–/–^ and 5 for *HNF1A-AS1*^intestine^^+/–^). Clustering by DSS and by genotype is also observed when including all samples with and without DSS exposure (Supplemental Figure 8D). In addition, α-diversity, a measure of within-sample variance, was significantly different between the groups (Figure 7N). In total, 459 microbial ASVs showed significant differential abundance (FDR ≤ 0.1) between *HNF1A-AS1*^+/+^ and *HNF1A-AS1*^intestine^^–/–^ at baseline prior to DSS administration (day 1; Supplemental Figure 8B). The PCoA plot that included all groups prior to DSS (day 1) and at day 6 and 10 of the experiment indicated the effect of DSS in all 3 groups on PC2 (Supplemental Figure 8, C–E), but *HNF1A-AS1*^intestine^^–/–^ samples that were taken before and after DSS seemed more intermixed (Supplemental Figure 8, D–F). Comparing unweighted UniFrac distances between samples of the same mice before and after DSS intake indicated that *HNF1A-AS1*^intestine^^–/–^ mice had the smallest microbial community change caused by DSS, which was significantly lower than that seen in *HNF1A-AS1*^+/+^, and with a similar trend seen when comparing to *HNF1A-AS1*^intestine^^+/–^ (Supplemental Figure 8F).

## Discussion

The human genome encodes thousands of lncRNAs (40), many of which are involved in a variety of key cellular processes (41) and implicated in human disease (7). In light of the increasing prevalence of UC, CD, and celiac, and the diagnosis of these chronic diseases at a relatively young age overall affecting many patients’ lives, we opted to highlight lncRNAs that are linked with these diseases as a first step to prioritize potential lncRNAs for preclinical functional analyses and as potential disease targets for interventions. We generated a comprehensive list of lncRNAs expressed in the small and large intestine, and we defined dysregulated lncRNAs in UC, CD, and celiac disease using independent test and validation cohorts from 696 mucosal mRNA-Seq samples that were reanalyzed uniformly starting from the raw data. Such a comprehensive platform can broadly benefit the research community to further expand the study of lncRNA functions (28) R Shiny web interface at https://tzipi.shinyapps.io/lncRNA_gut/ This method revealed 37 lncRNAs and 817 protein-coding genes dysregulated in celiac disease, CD, and UC. We characterize lncRNAs linked with UC severity and outcome in response to standardized initial therapy in PROTECT to further prioritize lncRNAs linked to nonresponse to available therapies, as potential targeting of these lncRNAs may facilitate better outcomes in conjunction with currently available therapies. We employed several methods to prioritize UC lncRNAs, including WGCNA to identify central lncRNAs within modules linked to clinical features of disease severity and prospective outcome, and we attempted to infer functionality using the better annotated coexpressed protein-coding genes.

Predicting the modes of action of lncRNAs is difficult because they are poorly characterized, their functional domains have not yet been fully established, and they seem to affect diverse cellular functions that rarely operate through a common mechanism of action. We propose several prioritization approaches for pinpointing functional lncRNAs based on their clinical disease context. We first employed an unbiased transcriptomics approach in test and validation treatment–naive cohorts, resulting in a comprehensive list of expressed and dysregulated lncRNAs in either UC, CD, or celiac. Prioritized lncRNAs included Morrbid lncRNA (also known as *MIR4435-2HG*), which was found to tightly control the lifespan of granulocytes (42); *IFNG-AS1* (also termed NeST) required for inducible IFN-γ synthesis in CD8^+^ T cells to control microbial susceptibility (10); *CEROX1* (also known as RP11-161M6.2), whose ortholog in mice was suggested to regulate mitochondrial complex I catalytic activity (14); and *LINC01272*, which was localized to leukocytes in the lamina propria of CD mucosal ileal biopsies (8) (Supplemental Data 4). *H19*, also prioritized by our analyses, was linked to epithelial proliferation and mucosal regeneration (43); *GATA6-AS1* (44) was shown to be expressed in gut epithelia and regulate epithelial renewal; and reduction of *ANRIL* (*CDKN2B-AS1*) was previously linked to IBD and barrier functions (45). We highlight lncRNA expression along the GI tract, demonstrating that lncRNAs have a more location-specific expression in comparison with protein-coding genes. It is possible that lncRNA expression along the GI tract evolved to guide the execution of location-specific functions — for example, in the small versus large intestine — as regulatory sequences including those linked with promoters and lncRNAs are known to evolve rapidly due to more relaxed structure-function constraints (46).

We utilized coexpression network analysis (WGCNA) to define lncRNA gene coexpression modules and the central hub within modules (lncRNAs with higher module membership [MM] score) using clinical context related to UC in the PROTECT cohort. Module M1, which contained lncRNAs reduced in UC (i.e., *GATA6-AS1*, *CDKN2B-AS1*, and *HNF1A-AS1* expressed by epithelial cells) was significantly associated with poorer UC course, while M5, which includes *LINC01272*, showed significant association with a poorer outcome of no W4R (*P* = 0.008). The intersection of M1 and M5 lncRNAs modules and lncRNAs plus protein-coding gene modules, to prioritize the lncRNAs more likely to be functional and to infer potential function from the better annotated protein-coding genes, indicated that 67% lncRNA from M1 (i.e*.*, *GATA6-AS1*) were included in the blue module enriched for mitochondrial TCA and lipid metabolism, which are known to affect inflammation and tissue damage in IBD (21, 47, 48). In total, 53% and 22% of the lncRNAs in M5 were included in the brown (i.e*.*, *AGAP2-AS1* and *RASSF8-AS1*) and black (i.e*.*, *LINC01272*) modules showing the strongest enrichment to stromal cells and adhesion, and to myeloid innate response respectively. These results are consistent with our prior characterization in ileal CD, where we highlighted the reduction of *HNF4A-AS1* linked with epithelial metabolic functions and induction of *LINC01272* linked with activation of myeloid immune signature (8). Finally, we defined significantly differentially expressed lncRNAs in severe versus mild UC cases and further prioritized those that are linked with disease course using a RF classifier and tested for significant associations with the paired gut microbiome samples. Microbial organisms and products that affect host mucosal barrier function and aberrant immune responses to commensal microbes are likely to contribute to gut inflammation (49). A previous study already demonstrated that a lncRNA-based tissue expression prediction model successfully identified different gnotobiotic mice from conventional and germ-free mice, and also discriminated mice harboring transplanted microbes from fecal samples of mice or zebra fishes, overall showing that lncRNA expression profiles in intestinal tissues can discriminate between different types of bacteria (50). Another study compared tissue specificity between germ-free conditions in the absence of gut microbiota and in mice raised in conventional conditions, showing different lncRNA signatures in the small and large intestine (51). Our data highlight significant positive associations between *GATA6-AS1* expression and *Blautia* (ASV09918) and a negative association between *GATA6-AS1* and Enterococcus (ASV13969). A higher abundance of Streptococcus (ASV12252) and Veillonella (ASV13143) taxa were previously linked to IBD in a large meta-analysis (52), and in our analysis, those showed a positive association with *LINC01272*. These are intriguing links between microbial abundance and mucosal lncRNA expression, and future studies will need to assess whether and how microbial signals contribute to the regulation of lncRNAs expression. Reduction of *HNF1A-AS1* in both mice and humans was linked with more severe colitis. *HNF1A-AS1* was within the 45 lncRNAs that showed similar dysregulation in all 3 diseases (Figure 2) — UC, CD, and celiac — and was within gene expression modules related to UC outcome in humans. We now show that specific reduction in *HNF1A-AS1* in mouse gut epithelia showed higher sensitivity to DSS-induced colitis, which was coupled with alteration in the microbial community. The gut microbiota composition can influence DSS outcome (53), and therefore as a limitation of this, it is not clear if the lack of *HNF1A-AS1* in the gut epithelium or the lack of *HNF1A-AS1* affecting the microbiota is linked with the colitis susceptibility. This concept, as well as additional controls and comparison with the reduction of other lncRNAs, should be further addressed in future work. Recent work showed that *HNF1A-AS1* (HASTER) is a *cis*-acting transcriptional stabilizer of *HNF1A* in pancreatic and liver cells (54), but the role of *HNF1A-AS1* in the intestine was not yet studied. Together, these observations suggest that *HNF1A-AS1* has a role in mucosal regeneration, and its lower expression in epithelia is linked with more severe mucosal injury. Mucosal damage leads to a leaky epithelial barrier, which promotes the exposure of deeper layers of the mucosa to intestinal microbial antigens, introducing them to the immune cells, which may lead to disturbed homeostasis, and these changes may be linked with the alteration in the gut microbial composition.

A notable strength of our study was the use of large, well-designed and characterized cohorts of treatment-naive patients, free from confounding variables of previous therapy. Other strengths include independent validation for the disease DE in other cohorts and exemplifying cell-specific expression of some lncRNAs in isolated and single-cell intestinal epithelia. We applied several complementary prioritization methods to highlight lncRNAs related to disease and when available, as in the PROTECT UC cohort, also to clinical context related to severity and disease course. Considering these lncRNAs as future targets include specific advantages of cellular and tissue specificity, their potential regulatory roles, and the fact that they are readily targetable by RNA therapeutics. Furthermore, the potential targeting of these lncRNAs linked to less favorable outcomes with available treatments may improve the outcome of nonresponders. Limitations include the use of treatment-naive samples, which are likely to be inflamed, but inflammation is the hallmark of these diseases and persistent inflammation not responding to currently available therapy drives the quest for identifying new interventional targets. Other limitations include the use of polyA selection in the library preparation, which captures most but not all lncRNAs, although nonpolyadenylated RNAs are generally highly unstable; the relatively small size of the SOURCE CD and SEEM celiac cohorts that were processed similarly to the PROTECT cohort that was used here as the test cohorts; and the lack of experimental validation of the functions of many of the prioritized lncRNA, which will complement the informatics analyses in future work.

### Conclusion.

We present a comprehensive attempt to characterize expressed and dysregulated lncRNAs in the human small and large intestines, linking lncRNAs to UC, CD, and celiac diseases using test and validation cohorts reanalyzing 696 mucosal biopsies. We revealed specific lncRNAs that are linked to UC severity and outcome and suggested ways to prioritize lncRNAs for further exploration. These include lncRNA expressed by epithelial cells linked to cell metabolism and lncRNA implicated in proinflammatory stromal and myeloid cell function. lncRNAs can be used as biomarkers for diagnosis, severity, and outcome but also as potential targets to improve outcomes.

## Methods

Supplemental Methods are available online with this article.

### Cohorts

PROTECT UC (21, 31) (206 UC and 20 Ctl cases) and SEEM celiac (22) (17 celiac cases with Marsh 3 and 25 controls) were described previously. Sheba Medical Center IRB approved the SOURCE cohort (18 CD and 25 controls) protocol. Informed consent was obtained. Validation cohorts included the published RISK cohort with baseline CD ileal (213 CD and 47 controls) and the UC rectal (43 UC and 55 controls) data sets (19, 21) and the celiac cohort (PRJNA528755, 12 celiac and 15 controls; ref. 25). Cohorts and cases are summarized in Supplemental Table 1.

#### RNA extraction and RNA-Seq analysis.

Differential expression and mRNA-Seq analyses were performed within each cohort including SOURCE, the already published PROTECT, RISK, SEEM, and celiac cohorts. Results obtained from each cohort were cross-compared with the other cohorts as indicated. Out of these cohorts, the PROTECT (GSE109142), SOURCE (GSE199906), and SEEM (GSE159495) test cohorts were experimentally processed similarly using similar technology in the same facility; mucosal biopsies were placed in RNAlater (Invitrogen) and kept frozen in –80°C. RNA extraction used Qiagen AllPrep RNA/DNA Mini Kit, and PolyA-RNA selection, fragmentation, cDNA synthesis, adaptor ligation, TruSeq RNA sample library preparation (Illumina), and paired-end 75 bp sequencing. Transcriptomics raw FATSQ files of all studies were computationally processed uniformly using the same pipeline. Reads were quantified by kallisto (55). mRNA genes with transcripts per million (TPM) values above 1 in at least 20% of the samples were used. Differential gene expression was performed using DESeq2 (56). WGCNA identified modules of coexpressed genes (32).

### Microbiome analysis

PROTECT 16S rRNA reads were processed using QIIME2 (57). Differentially abundant ASVs between patients with mild and severe UC were identified (nonparametric rank mean test, FDR < 0.1). HAllA testing identified correlations between lncRNAs and ASVs.

### Mouse models

*Haster* (*HNF1A-AS1*) LoxP C57BL/6J mice (54) were provided by the Jorge Ferrer group from the Barcelona Institute of Science and Technology (Barcelona, Spain) and the Imperial College London (London, United Kingdom). C57BL/6, Villin-Cre mice were crossed with *Haster* (*HNF1A-AS1*) LoxP C57BL/6J mice (54) to generate *HNF1A-AS1*^intestine^^–/–^ (intestine-specific deletion of *HNF1A-AS1* promoter in both alleles) and *HNF1A-AS1*^intestine^^+/–^ (intestine-specific deletion of *HNF1A-AS1* promotor in one of the alleles) animals. Male *HNF1A-AS1*^+/+^ C57BL/6J mice, 8–9 weeks old (catalogs 2BL/606, 2BL/606) were purchased from Envigo Laboratories. To induce colitis, mice were administered drinking water supplemented with dextran sulfate sodium (molecular weight, 36,000–50,000; MP Biomedicals, 160110) for 5 days and were then allowed to recover by drinking unsupplemented water for the next 6 days. All procedures performed were in accordance with the Sheba Medical Center’s Guidelines for Animal Studies and approved by the Institutional Animal Ethics Committee (ethical approval code 006_b16947).

### Statistics

Statistics used for transcriptomics and microbiome were performed in R, and details are provided in Supplemental Methods. Spearman’s rank correlation was used for continuous variables and the Mann-Whitney *U* test for categorical variables, with Benjamini-Hochberg FDR correction. χ^2^ test was used to test for differences between categorical variables. *q* values account for multiple testing corrections. Schemes were generated using biorender.com.

### Study approval

Published PROTECT, SEEM, RISK, and celiac data sets were used. SOURCE was approved by the Sheba Medical Center IRB. Informed consent was obtained from all participants. Informed consent was obtained. Validation cohorts included the published RISK cohort with baseline CD ileal and the UC rectal data sets and a second celiac cohort.

### Data availability

All RNA-Seq data sets are in GEO: PROTECT (GSE109142), SOURCE (GSE199906), SEEM (GSE159495), RISK (rectal GSE117993, ileal GSE101794), and celiac cohort (PRJNA528755; ref. 25). An interactive platform of lncRNA expression in celiac disease, CD, UC, and along the GI tract — proximal small intestine (duodenum), distal small intestine (ileum), and the large intestine (rectum) — in controls can be found through the R Shiny web interface at https://tzipi.shinyapps.io/lncRNA_gut/ (28).

The mouse 16S amplicon sequencing data set was deposited at the National Center for Biotechnology Information as BioProject PRJNA930578.

The code is available on GitHub at https://github.com/Tzipisb/lncRNA_gut_disease (Supplemental Data 5).

## Author contributions

TB and KES equally contributed to the manuscript; TB performed a major part of the bioinformatics and KES most of the laboratory experiments. Both conceived and designed the study, analyzed the data, and wrote the first draft of the manuscript. YH conceived and designed the study, analyzed the data, acquired funding for the study, and wrote the first draft of the manuscript. JSH, LAD conceived and designed the PROTECT study, helped analyzed the current data, and participated in drafting the manuscript. AA, MBS, KLV, RK, NL, HAE, RH, GE, DC, CA, MS, AGJ, BW, CM, IB, SBH, IU AB, and JF generated and analyzed the data and participated in drafting the manuscript. MJR, AMG, TDW, DRM, BMB, SAA, SRM, RJX, and SK recruited patients, collected, and analyzed data and participated in drafting the manuscript. All authors had access to study data and approved the manuscript.

## Supplementary Material

Supplemental data

Supplemental data set 1

Supplemental data set 2

Supplemental data set 3

Supplemental data set 4

Supporting data values

## Version 1 06/01/2023

In-Press Preview

## Version 2 07/24/2023

Electronic publication
