# Supplementary material for: Mucosal transcriptomics highlight lncRNAs implicated in ulcerative colitis, Crohn’s disease, and celiac disease
Source: JCI Insight. 2023 Jul 24;8(14):e170181. doi: 10.1172/jci.insight.170181 (PMC10443795; doi:10.1172/jci.insight.170181)
Supplement: Supplemental data [file jciinsight-8-170181-s239.pdf]

**Mucosal transcriptomics highlight lncRNAs implicated in ulcerative colitis, Crohn disease, and celiac disease**

Tzipi Braun[1*], Katya E. Sosnovski[1,2*], Amnon Amir[1], Marina BenShoshan[1,2], Kelli L. VanDussen[3], Rebekah Karns[3], Nina Levhar[1,2], Haya Abbas-Egbariya[1,2], Rotem Hadar[1], Gilat Efroni[1], David Castel[1], Camilla Avivi[1], Michael J. Rosen[3,4], Anne M. Grifiths[5], Thomas D. Walters[5], David R. Mack[6], Brendan M. Boyle[7], Syed Asad Ali[8], Sean R. Moore[9], Melanie Schirmer[10], Ramnik J. Xavier[11], Subra Kugathasan[12], Anil G. Jegga[3,13], Batya Weiss[1,2], Chen Mayer[1,2], Iris Barshack[1,2]Shomron Ben-Horin[1], Igor Ulitsky[14], Anthony Beucher[15], Jorge Ferrer[15], Jeffrey S. Hyams[16], Lee A. Denson[3], Yael Haberman[1,2,3]

[1]Sheba Medical Center, Tel-Hashomer, affiliated with the Tel Aviv University, Tel Aviv, Israel.
[2]Faculty of Medicine, Tel Aviv University, Tel Aviv, Israel.
[3]Cincinnati Children's Hospital Medical Center, Department of Pediatrics, University of Cincinnati College of Medicine, Cincinnati, OH, USA.
[4]Center for Pediatric IBD and Celiac Disease, Department of Pediatrics, Stanford University School of Medicine, Stanford, CA USA.
[5]Hospital for Sick Children, Toronto, Canada.
[6]Children's Hospital of East Ontario, Ottawa, Ontario, Canada.
[7]Nationwide Children's Hospital, Columbus, OH, USA.
[8]Department of Pediatrics and Child Health, Aga Khan University, Karachi, Pakistan.
[9]Department of Pediatrics, University of Virginia, Charlottesville, VA, USA
[10]The Technical University of Munich, Munich, Germany
[11]Broad Institute of MIT and Harvard University, Cambridge, MA, and Massachusetts General Hospital, Harvard Medical School, Boston, MA, USA
[12] Emory University, Atlanta, GA, USA.
[13]Department of Computer Science, Cincinnati Children's Hospital Medical Center and the University of Cincinnati College of Engineering, Cincinnati, OH, USA
[14]Departments of Biological Regulation and Molecular Neuroscience, Weizmann Institute of Science, Rehovot, Israel.
[15]The Barcelona Institute of Science and Technology (BIST), 08003 Barcelona, Spain
[16]Connecticut Children's Medical Center, Hartford, CT, USA.

*Co-first authors

Corresponding Author:
Yael Haberman, MD, PhD (Yael.Haberman@sheba.health.gov.il and yael.haberman@cchmc.org)
Sheba Medical Center, 2 Derech Sheba, Tel-Hashomer, 52621, Israel.
Telephone number: 972-3-5302692

## Supplementary Material

**Supplementary Tables** *– page 3*
**List of supplementary datasets** *– page 7*
**Supplementary Figures** *– page 8*
**Supplementary Methods** *– page17*

## Supplementary Tables

**Suppl. Table 1:** cohorts used (main and validation). All main cohorts were sequenced using a similar pipeline.

| Disease (*Dx location) | *Test/ validation | Study | Cases (n) | Controls (n) | Cohort population | Sequencing platform | CCHMC Sequencing core | Coverage Median (IQR) | Reference | Data availability |
|---|---|---|---|---|---|---|---|---|---|---|
| Ulcerative colitis (Rectum) | Test | PROTECT | 206 | 20 | Pediatric | TruSeq polyA Paired-end mRNASeq | Yes | 43M (37, 52) | PMID: 30604764 | GSE109142 |
| | Validation | RISK | 43 | 55 | Pediatric | TruSeq polyA Single-end mRNASeq | Yes | 17M (12, 20) | PMID: 30604764 | GSE117993 |
| Crohn's Disease (Terminal ileum) | Test | SOURCE | 18 | 25 | Adult | TruSeq polyA Paired-end mRNASeq | Yes | 38M (34, 43) | unpublished | GSE199906 |
| | Validation | RISK | 213 | 47 | Pediatric | TruSeq polyA Single-end mRNASeq | Yes | 17M (15, 21) | PMID 25003194 | GSE57945 |
| Celiac (Duodenum) | Test | SEEM | 17 | 25 | Pediatric | TruSeq polyA Paired-end mRNASeq | Yes | 34M (33, 46) | PMID: 33524399 | GSE159495 |
| | Validation | Leonard et al | 12 | 15 | Adult | KAPA stranded mRNA-Seq | No | 49M (42, 58) | PMID: 30998704 | PRJNA528755 |
| | *All studies used fresh biopsies and were re-analyzed using a similar analytic pipeline (see methods). | | | | | | | | | |

**Suppl. Table 2:** PROTECT treatment naïve cohort and outcome

| | Ctl (n=20) | UC (n=206) |
|---|---|---|
| Age (Mean ± SD) | 13.9 ± 3.3 | 12.9 ± 3.2 |
| Sex M (%) | 9 (45%) | 112 (54%) |
| BMI z score (Mean ± SD) | 0.3 ± 1.6 | -0.26±1.32 |
| White | 17/20 (85%) | 204/206 (99%) |
| PUCAI score (range 0-85) | | |
| 10-30 (Mild) | - | 54 (26%) |
| 35-60 (Moderate) | - | 84 (41%) |
| ≥65 (Severe) | - | 68 (33%) |
| Mayo endoscopy subscore (range 0-3) | | |
| Grade 1 Mild | - | 27 (13%) |
| Grade 2 Moderate | - | 108 (52%) |
| Grade 3 Severe | - | 71 (34%) |
| Disease location | | |
| Proctosigmoiditis | - | 14 (7%) |
| Left-sided colitis | - | 25 (12%) |
| Extensive /Pancolitis / *Unassessable | - | 167 (81%) |
| Initial Treatment | | |
| Mesalamine | - | 53 (26%) |
| Oral or IV steroids | - | 153 (74%) |
| Oral steroids | - | 82 (40%) |
| IV steroids | - | 71 (34%) |
| Week 4 remission (PUCAI<10) | - | 105 (51%) |
| Week 52 CSFR | - | 75/206 (36%) |
| 3 Years colectomy | | 17/206 (8.3%) |

Unassessable: severe/fulminant disease at presentation and the clinician performed a flexible sigmoidoscopy for safety concerns. Data are mean ± SD, n (%), n/N (%) unless noted otherwise. PUCAI=Pediatric Ulcerative Colitis Activity Index.

| **Suppl. Table 3:** SOURCE treatment naïve cohort | | |
|---|---|---|
| | Ctl (n=25) | CD (n=18) |
| Age (Mean ± SD) | 35 ± 16 | 32 ± 11 |
| Sex M (%) | 12 (48%) | 11 (61%) |
| Ethnicity white (%) | 25 (100%) | 18 (100%) |
| <u>Disease location</u> | | |
| L1 | - | 12 (67%) |
| L2 | - | 0 (0%) |
| L3 | - | 6 (33%) |
| <u>Behavior status</u> | | |
| B1 | - | 15 (83%) |
| B2 | - | 2 (11%) |
| B3 | - | 1 (6%) |

| **Suppl. Table 4:** SEEM (GSE159495) treatment naïve cohort (biopsies from diagnostic endoscopy) | | |
|---|---|---|
| | Ctl (n=25) | Celiac (n=17) |
| Age (Mean ± SD) | 5.5 ± 2.2 | 8 ± 2.5 |
| Sex M (%) | 14 (56%) | 8 (47%) |

| **Table S5: Models that include bassline clinical or endoscopic severity and transcriptomics signals (summarize to PC1) from Figure 6 and suppl. dataset 4 including only the moderate-severe UC cases (n=153)** | | | | | | |
|---|---|---|---|---|---|---|
| Models for W4 with baseline clinical (PUCAI severity) | | | | | | |
| Model # | Outcome | Predictor | OR (95% CI) | P-value | AIC | AUC |
| 1 | Wk4 | PUCAI (baseline) | 0.966 (0.944, 0.989) | 0.0043 | 207.431 | 0.635 |
| | | | | | | |
| 2 | Wk4 | PUCAI (baseline) | 0.979 (0.955, 1.004) | 0.0937 | 198.955 | 0.698 |
| | | lncRNA PC1(explaining 27.8% of the variation) | 0.921 (0.874, 0.97) | 0.002 | | |
| | | | | | | |
| 3 | Wk4 | PUCAI (baseline) | 0.977 (0.953, 1.002) | 0.067 | 201.359 | 0.702 |
| | | Protein coding PC1(explaining 35.1% of the variation) | 0.972 (0.952, 0.992) | 0.006 | | |
| | | | | | | |
| 4 | Wk4 | PUCAI (baseline) | 0.977 (0.953, 1.002) | 0.0715 | 200.919 | 0.703 |
| | | LncRNA+Protein coding PC1(explaining 33.6% of the variation) | 0.973 (0.954, 0.992) | 0.005 | | |

| Models for W4 with baseline endoscopic severity (Mayo score) | | | | | | |
|---|---|---|---|---|---|---|
| Model # | Outcome | Predictor | OR (95% CI) | P-value | AIC | AUC |
| 5 | Wk4 | Endoscopic Mayo (baseline) | 0.433 (0.243, 0.773) | 0.004 | 207.502 | 0.624 |
| | | | | | | |
| 6 | Wk4 | Endoscopic Mayo (baseline) | 0.613 (0.328, 1.146) | 0.125 | 199.405 | 0.699 |
| | | lncRNA PC1 (explaining 27.8% of the variation) | 0.921 (0.873, 0.971) | 0.002 | | |
| | | | | | | |
| 7 | Wk4 | Endoscopic Mayo (baseline) | 0.583 (0.313, 1.087) | 0.089 | 201.841 | 0.701 |
| | | Protein coding PC1(explaining 35.1% of the variation) | 0.972 (0.952, 0.993) | 0.008 | | |
| | | | | | | |
| 8 | Wk4 | Endoscopic Mayo (baseline) | 0.59 (0.316, 1.101) | 0.097 | 201.408 | 0.702 |
| | | LncRNA+Protein coding PC1(explaining 33.6% of the variation) | 0.973 (0.954, 0.992) | 0.006 | | |

| Models for W52 with baseline clinical (PUCAI severity) | | | | | | |
|---|---|---|---|---|---|---|
| Model# | Outcome | Predictor | OR (95% CI) | P-value | AIC | AUC |
| 1 | WK52 | PUCAI (baseline) | 1.003 (0.98, 1.027) | 0.787 | 200.057 | 0.515 |
| | | | | | | |
| 2 | WK52 | PUCAI (baseline) | 1.017 (0.991, 1.044) | 0.211 | 194.155 | 0.62 |
| | | lncRNA PC1 (explaining 27.8% of the variation) | 0.930 (0.883, 0.98) | 0.0065 | | |
| | | | | | | |
| 3 | WK52 | PUCAI (baseline) | 1.013 (0.988, 1.04) | 0.303 | 196.972 | 0.618 |
| | | Protein coding PC1(explaining 35.1% of the variation) | 0.977 (0.957, 0.998) | 0.0291 | | |
| | | | | | | |
| 4 | WK52 | PUCAI (baseline) | 1.014 (0.988, 1.04) | 0.286 | 196.548 | 0.619 |
| | | LncRNA+Protein coding PC1(explaining 33.6% of the variation) | 0.978 (0.959, 0.997) | 0.0231 | | |

| Models for W52 with baseline endoscopic severity (Mayo score) | | | | | | |
|---|---|---|---|---|---|---|
| Model# | Outcome | Predictor | OR (95% CI) | P-value | AIC | AUC |
| 5 | WK52 | Endoscopic Mayo (baseline) | 0.728 (0.41, 1.293) | 0.279 | 198.953 | 0.544 |
| | | | | | | |
| 6 | WK52 | Endoscopic Mayo (baseline) | 0.963 (0.513, 1.807) | 0.906 | 195.735 | 0.625 |
| | | lncRNA PC1 (explaining 27.8% of the variation) | 0.943 (0.896, 0.993) | 0.0249 | | |
| | | | | | | |
| 7 | WK52 | Endoscopic Mayo (baseline) | 0.898 (0.482, 1.675) | 0.736 | 197.928 | 0.631 |
| | | Protein coding PC1(explaining 35.1% of the variation) | 0.982 (0.962, 1.003) | 0.089 | | |
| | | | | | | |
| 8 | WK52 | Endoscopic Mayo (baseline) | 0.91 (0.487, 1.699) | 0.766 | 197.612 | 0.633 |
| | | LncRNA+Protein coding PC1(explaining 33.6% of the variation) | 0.982 (0.963, 1.002) | 0.074 | | |

**Supplementary data**

**Suppl. Data 1**: DESeq2 differentially expressed lncRNAs and protein-coding genes, in UC, CD, and celiac test cohorts, with FC and FDR in validation cohorts, and a comparison across the 3 diseases.

**Suppl. Data 2**: expressed lncRNAs in control rectum, ileum, duodenum (used in Figure 3), expressed protein-coding in control rectum, ileum, duodenum

**Suppl. Data 3:** WGCNA with only lncRNA, WGCNA with protein-coding and lncRNA, functional annotation enrichment results for the WGCNA with protein-coding and lncRNA modules

**Suppl. Data 4:** Severe vs. mild (PUCAI) differentially expressed lncRNAs, Severe vs. mild (PUCAI) differentially expressed protein-coding genes, RF genes for predicting early (W4) and W52 SFR, differentially expressed ASVs between severe and mild UC (PUCAI), associations between lncRNAs and ASVs, associations between protein-coding genes and ASVs.

**Suppl. Data 5:** Supporting data values associated with the manuscript figures.

## Supplementary Figures

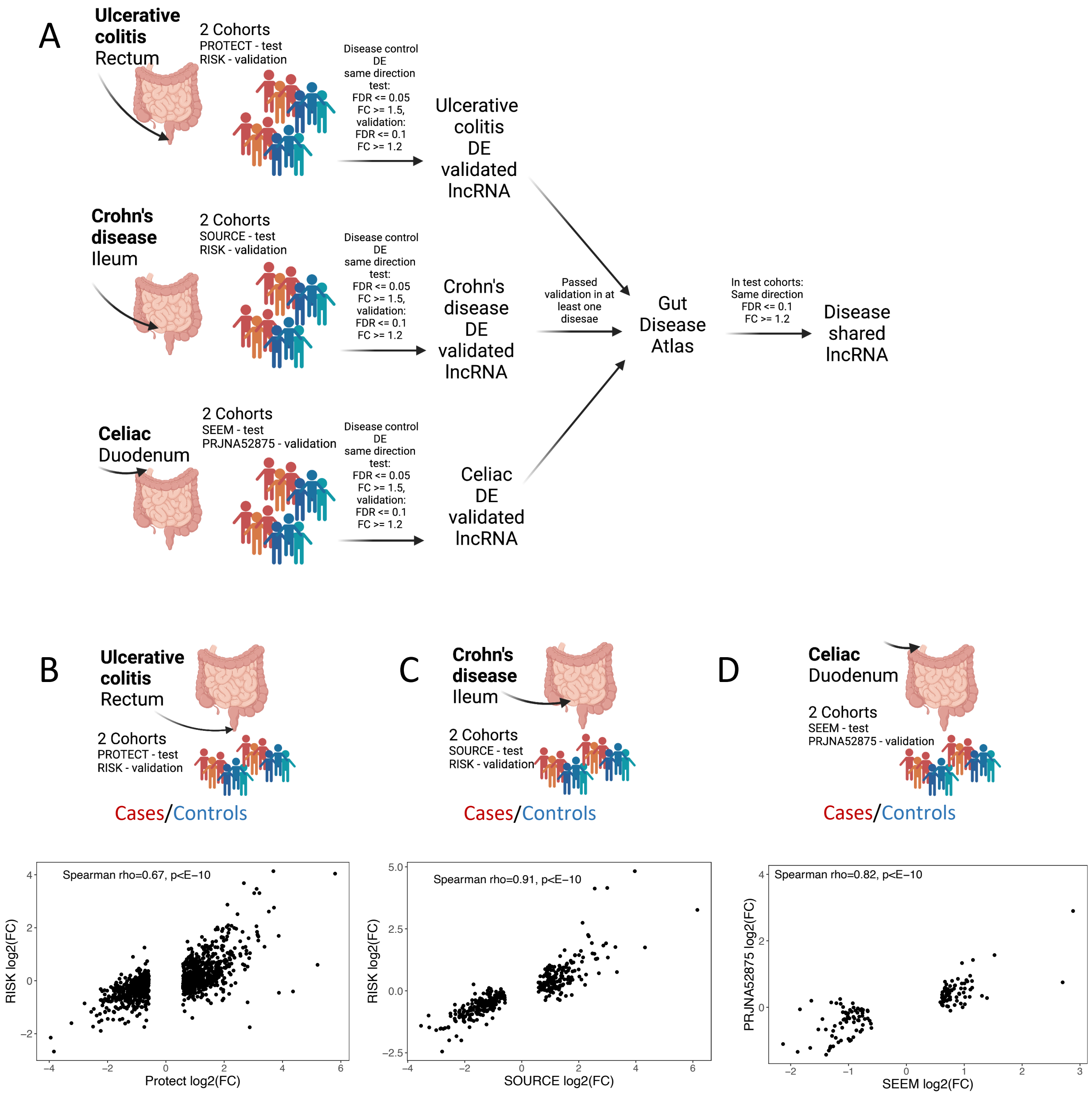


**Figure S1: Differential expression of deregulated lncRNAs in celiac duodenum, Crohn ileum, and UC rectum. A.** Scheme showing the process of identifying lncRNAs dysregulated in celiac duodenum, Crohn ileum, and UC rectum. **B.-D.** scatter plots of log2(FC) values calculated between cases and control samples, for lncRNAs that passed DE in the main cohorts, and log(FC) values in of the same lncRNAs in the validation cohorts, showing consistency between main and validation cohorts. Spearman's rho and p values as calculated between main and validation log2(FC) values are shown. **B.** UC rectum samples, main - PROTECT, validation - RISK. **C.** Crohn's disease ileum samples, main - SOURCE, validation - RISK. **D.** Celiac duodenum samples, main - SEEM, validation - PRJNA52875. Schemes were generated using biorender.com

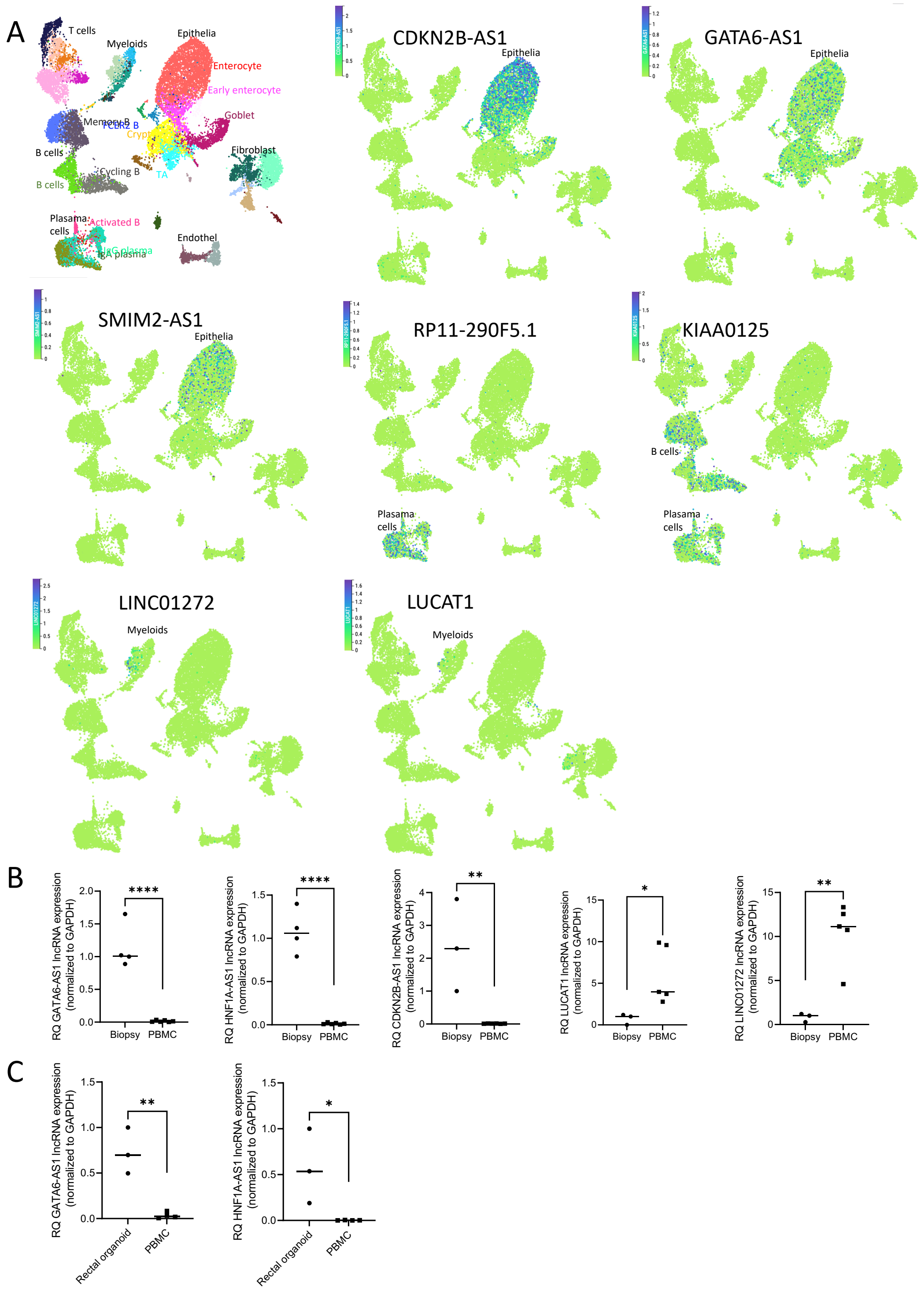


**Figure S2: Cell-specific trends in gene expression of the lncRNAs. A.** CD single cell was used for validation of lncRNA expression(1) (https://www.gutcellatlas.org); *GATA6-AS1, SMIM2-AS1*, and *CDKN2B-AS1* in gut epithelia, and *RP11 290F5.1* and *KIAA0125* in plasma cells and B cells. **B.** Indicated lncRNA expression in ileal biopsies and in peripheral leukocytes (peripheral blood mononuclear cells, PBMC) in control cases. Graphs with individual RQ values are shown normalized to GAPDH. T test ****p<0.0001, **p<0.01, *p<0.05**. C.** GATA6-AS1 and HNF1A-AS1 expression in epithelial is shown in differentiated rectal organoids from control with no detected expression in PBMC. Graphs with individual RQ values are shown normalized to GAPDH. T test **p<0.01, *p<0.05**.**

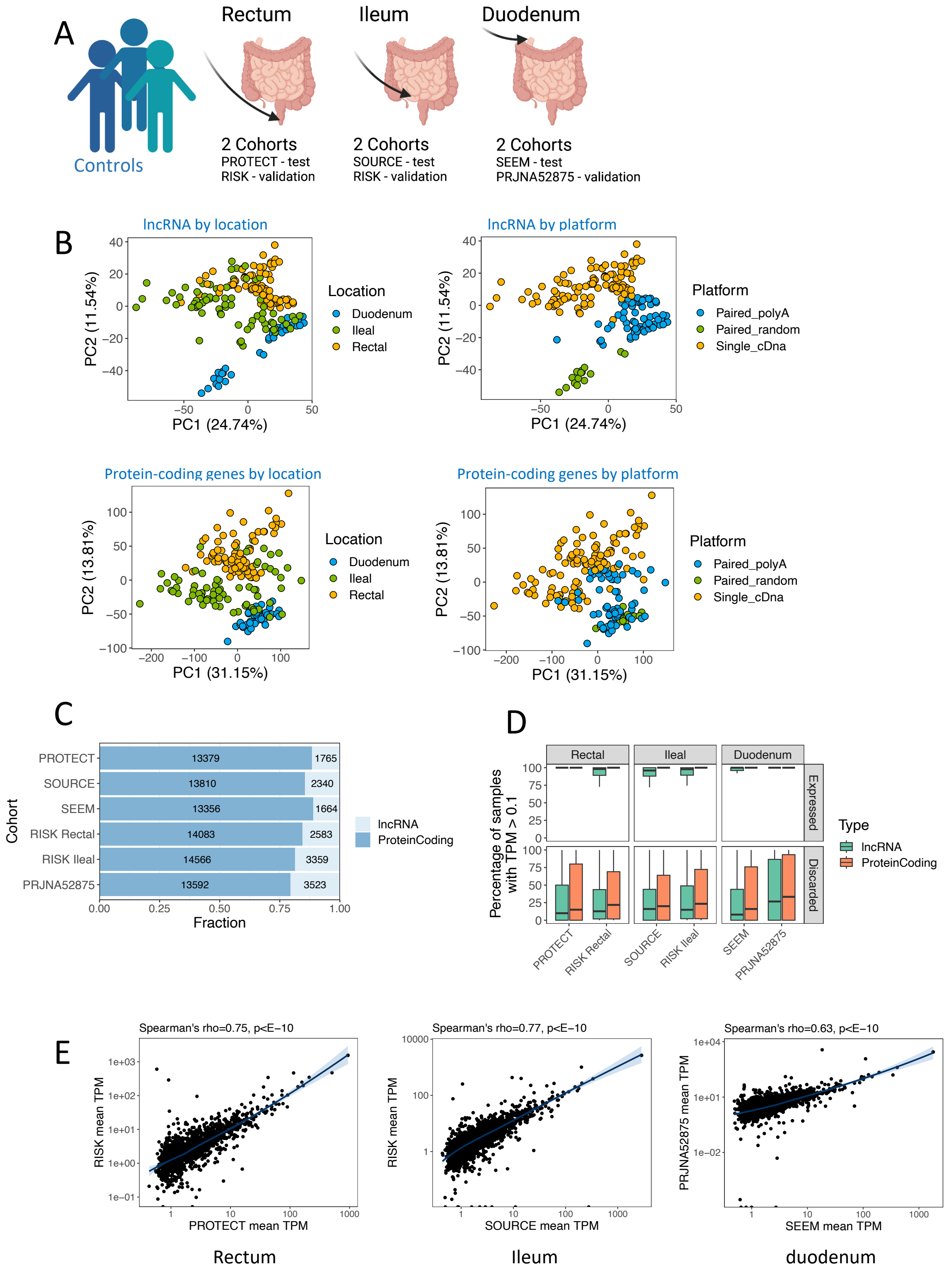


**Figure S3: lncRNA characterization in duodenum ileum and rectal control samples. A.** Scheme of the control samples, originating from 3 main and 3 validation cohorts – 2 rectum cohorts (PROTECT, RISK), 2 ileum cohorts (SOURCE, RISK) and 2 duodenum cohorts (SEEM, PRJNA52875). **B.** PCA figures of all main and validation control samples, colored by location (left) and platform (right). All 2933 lncRNAs (up), or 14377 protein-coding genes (down) expressed in the samples included in this analysis were used (TPM of over 1 in at least 20% of samples) **C.** For each cohort, the fraction of

lncRNA and protein-coding genes that passed expression filtering is marked on the x-axis, and the actual number of genes is written within the bar. **D.** Boxplots showing the percentage of samples that had a TPM value higher than 0.1, for each lncRNA and protein-coding gene. Genes were stratified to those that passed expression filtering by the criteria used in our main analysis (TPM > 1 in at least 20% of samples, see Methods), and that did not and were discarded from our analysis. Most genes that passed expression filtering appear to be highly expressed when using the lower cutoff of 0.1 TPM, while the discarded lncRNAs appear to have a similar expression to the discarded protein-coding genes, indicating that the observed lncRNA tissue specificity is not only due to lncRNA low expression. **E.** Scatter plots showing the average TPM values per gene of main and validation control samples, using genes that passed validation in main cohorts. Left = Rectum, Middle = Ileum, Right = Duodenum. Spearman's correlation values are noted above the figures.

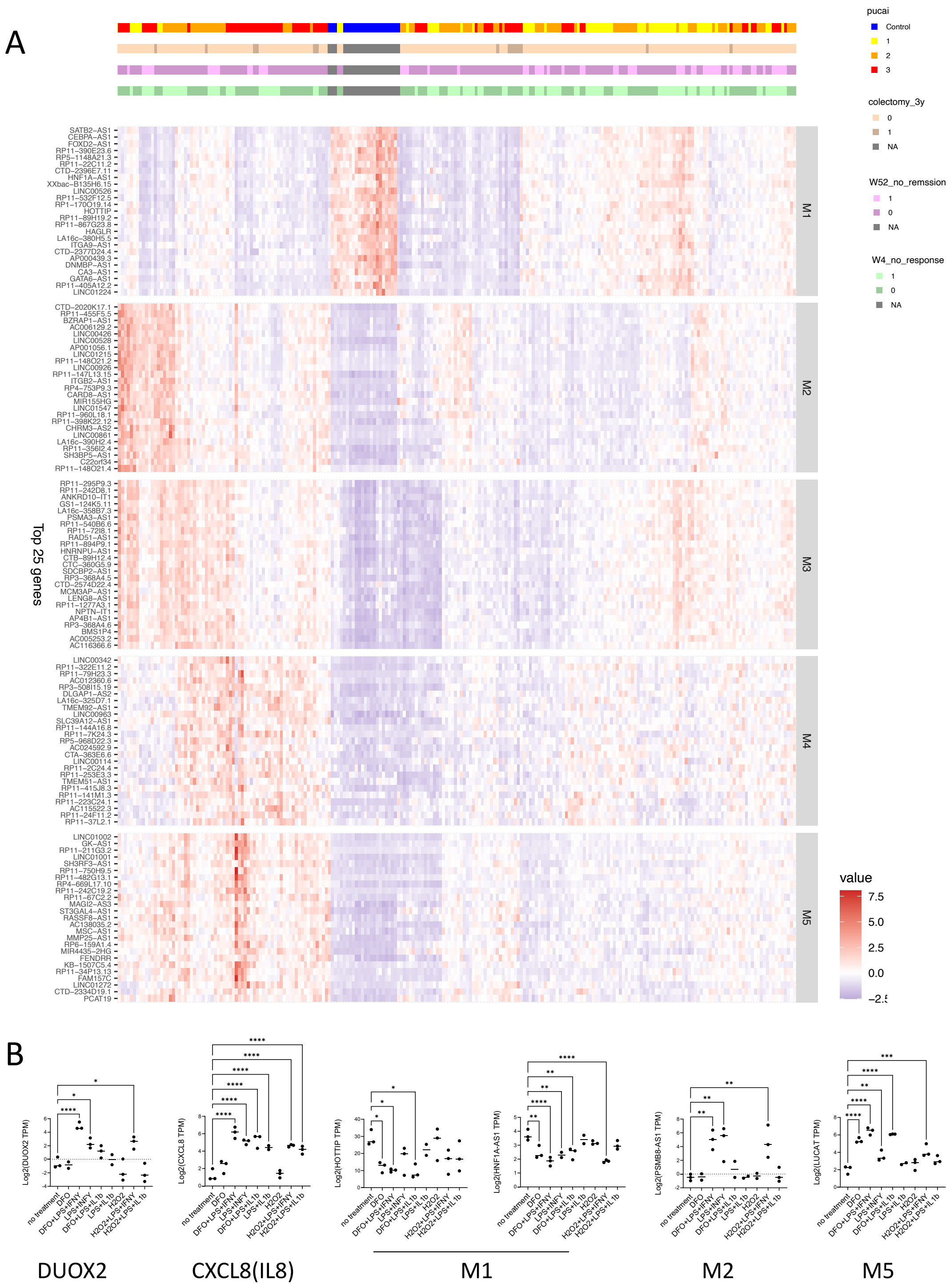


**Figure S4: Top 25 lncRNA in each WGCNA module from Figure 4G. A.** Heatmap showing z-score values of each sample of the top 25 lncRNAs in each WGCNA module, prioritized by MM values. Samples PUCAI clinical severity, 3-year colectomy, week 52 no CS free remission, and week 4 no response status are noted in color. Arrow indicates *GATA6-AS1*. **B.** Expression (reads per million) of DUOX2 and CXCL8 (IL8) and the indicated lncRNAS in HT-29 cells without and with 100uM DFO, 100uM DFO with 100ng/ml LPS plus 40ng/ml IFNγ, 100ng/ml LPS plus 40ng/ml IFNγ, 100uM DFO with 100ng/ml LPS plus 25ng/ml IL1b, 200uM H2O2, 200uM H2O2 with 100ng/ml LPS plus 40ng/ml IFNγ, and 200uM H2O2 with 100ng/ml LPS plus 25ng/ml IL1b**.**

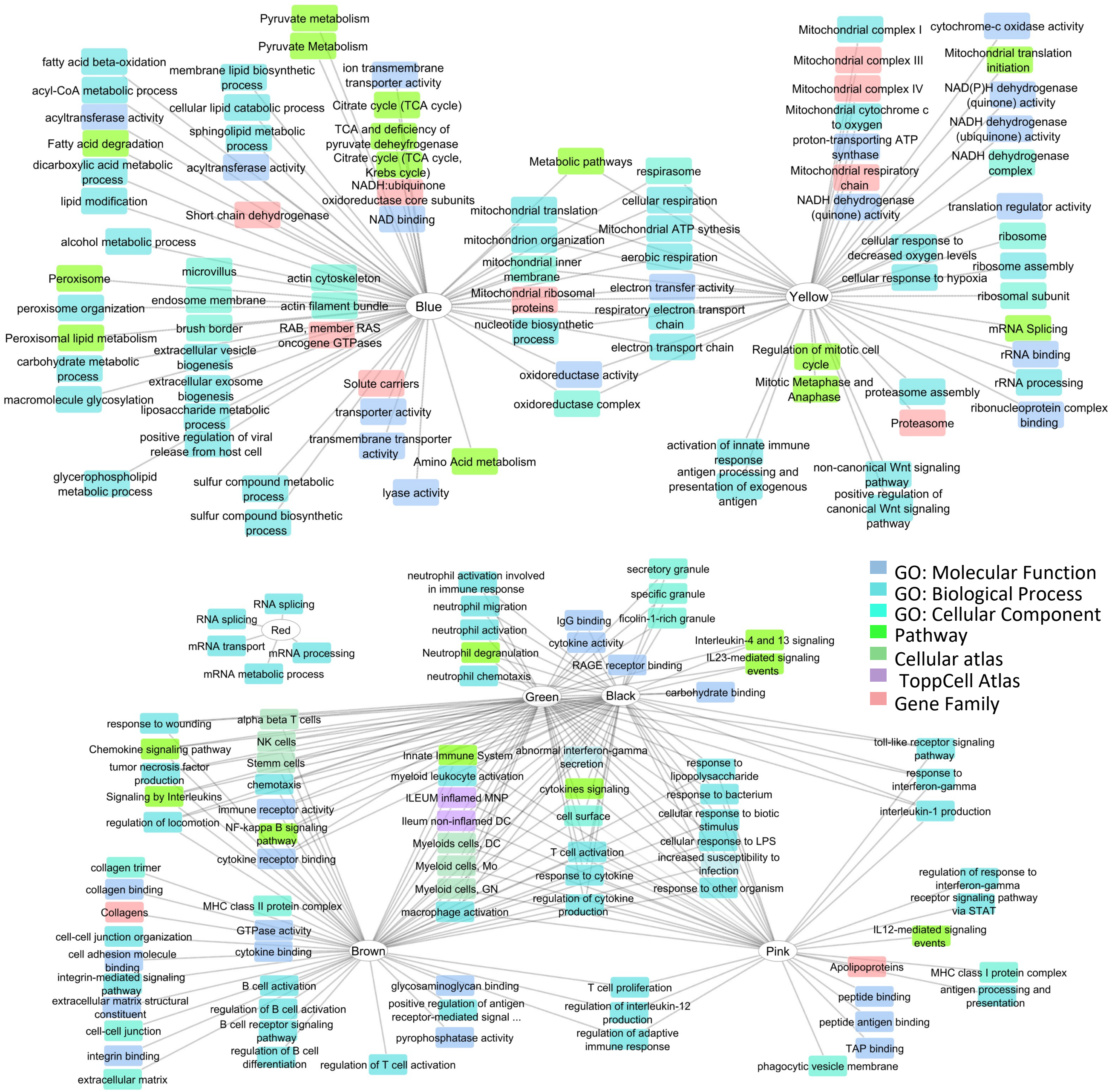


**Figure S5: More detailed functional annotation enrichment of the WGCNA from Figure 5.** Toppgene/Toppcluster functional annotation enrichment of protein-coding genes within each module. Annotations' origin database is marked by a specific color. Shown annotations were manually curated from the full list which can be found in Dataset S1. Cytoscape(2) is used for visualization.

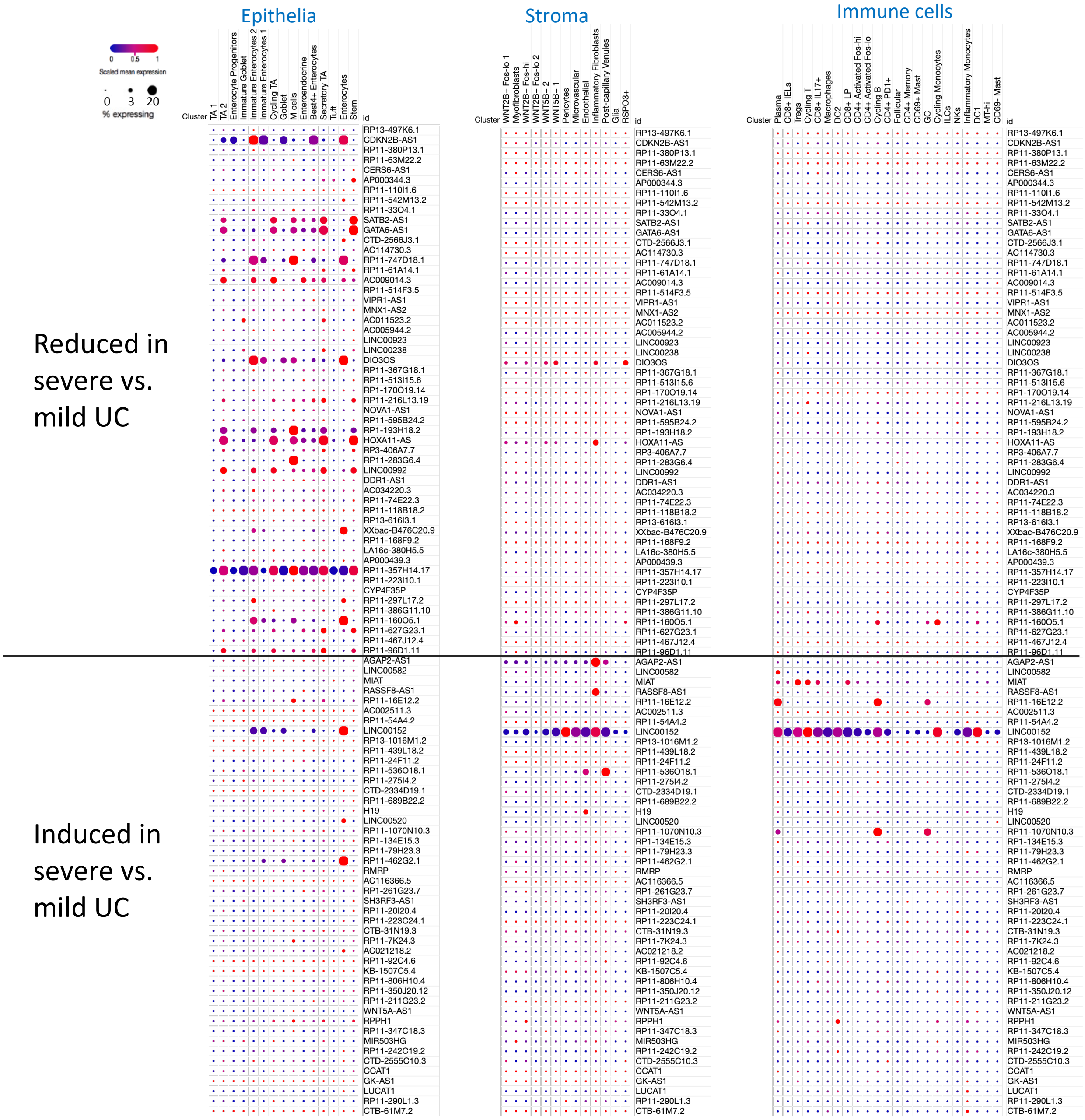


**Figure S6: The majority of the reduced lncRNAs between severe and mild UC_show epithelial specific expression.** Cellular expression of lncRNAs that were dysregulated between severe and mild UC and were expressed in the adult colon single-cell RNA-seq data(3). The size and color of the dots are proportional to the percentage of cells expressing the gene and the normalized expression level, respectively.

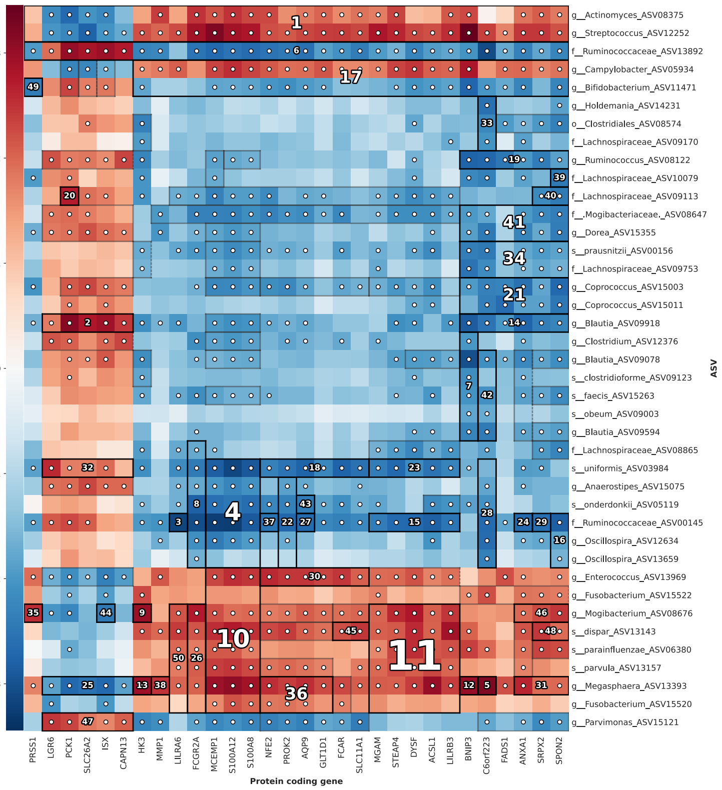


**Figure S7: Heatmap summarizing the association between protein coding gene expression and microbial ASV abundance.** Heatmap summarizing the association between protein coding gene expression and microbial ASV abundance that are linked with severity (Figure 6 and Dataset S4), using hierarchical All-against-All significance testing (HAllA), with FDR<0.1.

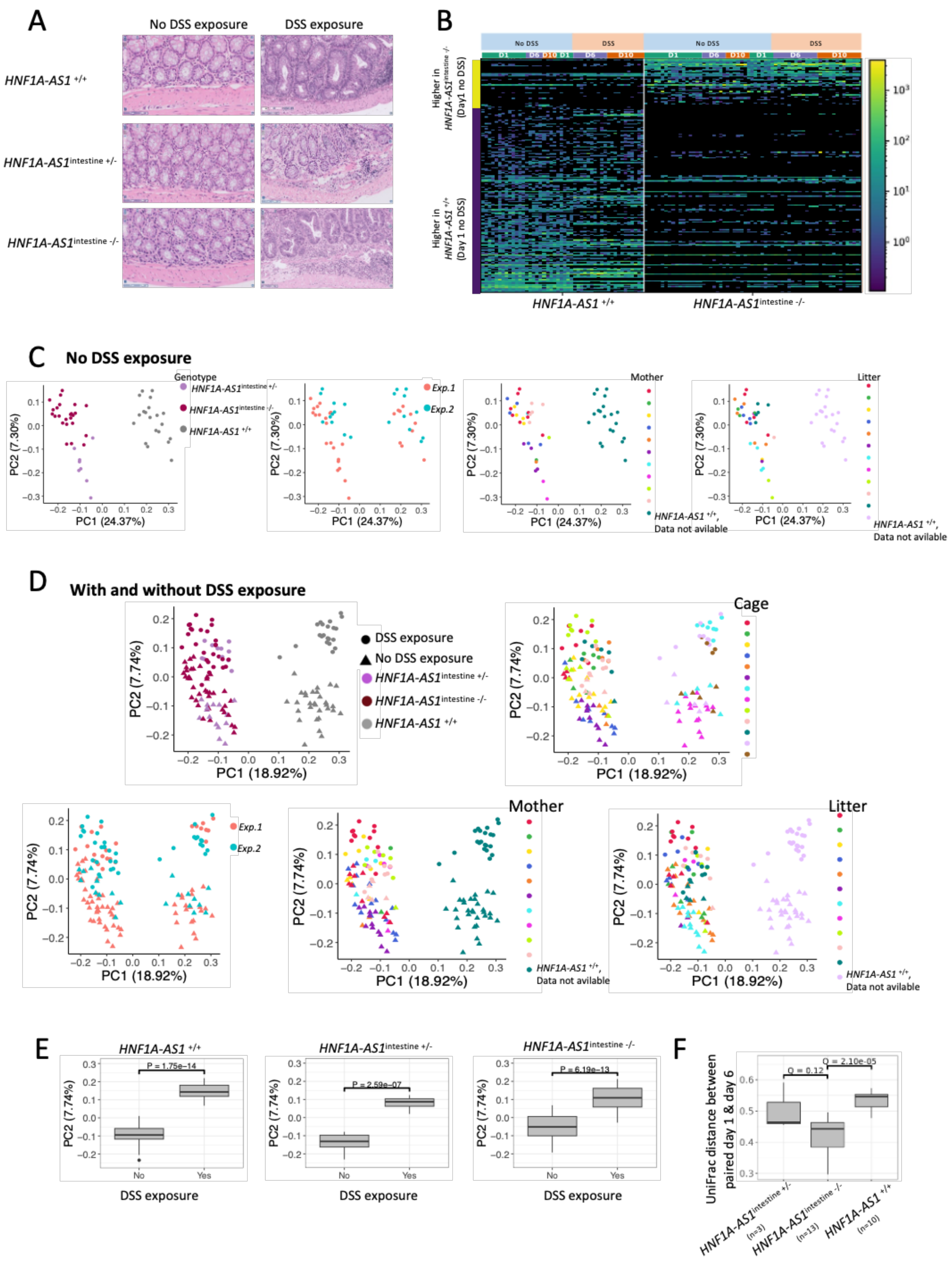


**Figure S8: Effect of DSS exposure on gut bacteria. A.** Representative histology with and without exposure to DSS at the end of the experiment. **B.** Heatmap of 459 differently abundant amplicon sequence variants (ASVs) bacteria in *HNF1A- AS1*$^{\text{intestine -/-}}$ and *HNF1A-AS1*$^{+/+}$ (FDR<0.25) showing the two

groups with/without exposure to DSS treatment. **C-D.** PCoA plot of mice bacteria, with/without DSS exposure as indicated, and colored by mice group, cage, experiment (1 or 2), mother, and litter as indicated. **E.** Box plots of PC2 values in *HNF1A- AS1*$^{\text{intestine -/-}}$, *HNF1A- AS1*$^{\text{intestine +/-}}$, and *HNF1A-AS1*$^{+/+}$ groups with/without DSS exposure. **F.** Box plots of unweighted UniFrac distance of the same mice, calculated between two Day 1 and Day 6 (with DSS) timepoints, within the 3 groups. P. values were calculated using Mann-Whitney test, and q. values with Benjamini-Hochberg FDR correction.

## Supplementary Methods

### Cohorts

PROTECT cohort (Test cohort 1) was described previously(4, 5). PROTECT is a multicenter inception cohort study including treatment naïve children aged 4–17 years with a diagnosis of UC. Disease extent, clinical activity at diagnosis (PUCAI range 0−85), Mayo endoscopic scope (grade 1-3), and total Mayo score (range 0−12) were captured. PUCAI less than 10 denoted inactive disease or remission, 10−30 denoted mild disease, 35−60 denoted moderate disease, and 65 or higher denoted severe disease. A central pathologist blinded to clinical data examined a single rectal biopsy. Depending on initial PUCAI score, patients received initial treatment with either mesalamine (mild disease), or corticosteroids (moderate and severe disease), with some physician discretion allowed. Week 4 (W4) remission outcome defined as PUCAI < 10 without additional therapy or colectomy, Week 52 as CS-free remission (SFR) with no therapy beyond mesalazine, and occurrence of colectomy within 3 years were recorded. Rectal mucosal biopsies from a representative sub-cohort of 206 PROTECT UC patients and 20 age and gender matched non-IBD controls underwent high coverage transcriptomic profiling using Illumina RNAseq. The 20 controls at Cincinnati Children's Hospital Medical Center were included in the current analyses as non-IBD controls after clinical endoscopic, and biopsies evaluation demonstrated no histologic and endoscopic inflammation.

SOURCE cohort (Test cohort 2) included 18 treatment naïve Crohn disease and 25 non-IBD controls from the Sheba Medical Center. Age, gender, endoscopic findings, diagnostic calprotectin, and CRP were recorded. All participants were White. Sheba Institutional Review Board approved the protocol and safety monitoring plan. Informed consent was obtained for each participant.

SEEM celiac cohort (Test cohort 3) was described previously(6) and included 17 celiac subjects and 25 controls from Cincinnati Children's Hospital Medical Center (CCHMC). Controls were subjects who were investigated for various gastrointestinal symptoms including abdominal pain, but had normal endoscopic and histologic findings. Celiac disease diagnosis was based on previously described algorithms(7) including positive IgA autoantibodies against tissue transglutaminase (anti-TTG) and histologic features.

Validation cohorts included the RISK cohort, a treatment naïve pediatric IBD cohort that was used to validate the baseline CD ileal dataset and the UC rectal gene expression dataset(5, 8) and the previously published celiac cohort(9).

Epithelial and single cell datasets. Isolated epithelial dataset included reanalyzes of a publicly available adult human colon UC single cell data set, which contains 366,650 cells from 18 UC and 12 healthy adult colons, to examine cell-specific trends in gene expression(3). A second CD single cell was used for validation of lncRNA expression in specific cell types(1) (https://www.gutcellatlas.org).

**RNA extraction and RNA-seq Analysis**. All transcriptomics analyses started from the raw FATSQ files using a similar pipeline. Differential expression and mRNA analyses was performed within each cohort including the already published PROTECT, RISK, SEEM, and celiac cohorts. Results obtained from each cohort were cross compared with the other cohorts as indicated. PROTECT (GSE109142), SOURCE (GSE199906), and SEEM (GSE159495) test cohorts were processed and sequenced using the same laboratory pipeline, sequencing library at the Gene and Protein Expression and Bioinformatics cores of the National Institutes of Health (NIH)-supported Cincinnati Children's Hospital Research Foundation Digestive Health Center. RNA was isolated from biopsies obtained during diagnostic colonoscopy using the Qiagen AllPrep RNA/DNA Mini Kit. PolyA-RNA selection, fragmentation, cDNA synthesis, adaptor ligation, TruSeq RNA sample library preparation (Illumina, San Diego, CA), and paired-end 75bp sequencing was performed. Median read depth in PROTECT was ~43M (37-52M IQR), in SOURCE 39M (31-46M IQR, and ~34M (33-46M IQR) in SEEM. RISK treatment naïve pediatric patients' rectal biopsies and ileal biopsies (rectal GSE117993, ileal GSE101794) were used as validation cohorts for UC and CD respectively using single end 75bp mRNA sequencing(5, 8). Those biopsies had similar RNA extractions at Cincinnati Children's and were also sequenced at the (NIH)-supported Cincinnati Children's Hospital Research Foundation Digestive Health Center. Another celiac cohort (PRJNA528755(9)) was used for validation, this cohort used KAPA stranded mRNA-Seq.

Reads were quantified by kallisto(10) version 42.5 using Gencode v24 as the reference genome. Kallisto output files were summarized to gene level using R package tximport version 1.12.3(11). protein coding genes and lncRNAs with Transcripts per Million (TPM) values above 1 in at least 20% of the samples were used in downstream analysis. Differentially expressed genes between cases and controls, or mild and severe UC had fold change (FC) $\geq$ 1.5 and false discovery rate (FDR) $\leq$ 0.05 using R package DESeq2 version 1.30.1(12).

Principal Coordinates Analysis (PCA) was performed to summarize variation in gene expression between patients, and principal components (PC) values were extracted for downstream analyses. Clinical features data were correlated to PC values using Spearman's correlation.

**Similar and specific genes between diseases and locations**

Identifying a validated specific disease signal for each disease and location: For each of the 3 main cohorts (PROTECT, SOURCE, SEEM) and the validation cohorts (RISK, Leonard et al.), differentially expressed genes between cases and controls was calculated using DESeq2. Within each disease and location, genes were considered validated if they: 1. Were significantly different between disease and control in the main cohort, with FC $\geq$ 1.5 and FDR $\leq$ 0.05. 2. Changed in the same direction in both main and validation cohorts. 3. Were significantly different between disease and control in the validation cohort, with FC $\geq$ 1.2 and FDR $\leq$ 0.1.

Identifying similar genes between diseases and locations: A list of all genes that passed validation in at least one disease was compiled, as our gut disease "atlas", shown in table S4. Within this atlas, a gene is considered similar between diseases and locations if it: 1. Changes in the same direction in all main cohorts. 2. Is significantly different between disease and control in all main cohorts, with FC $\geq$ 1.2 and FDR $\leq$ 0.1. This analysis was performed separately for lncRNA and protein-coding genes.

**Random forest ROC**

The random forest analysis was performed in R package randomForest(13) version 4.6-14 with default parameters, using 500 trees. For each disease and location, random forest was trained on control and disease samples from the main cohort, and tested on the validation cohort. Main cohort expressed genes were used, and main samples were randomly chosen to create equal sized disease and control groups. The randomForest package out-of-box estimate of error rate was used to estimate the main cohorts' classification, and the predict function for the validation cohorts. For the UC outcome analysis (week 4 remission and week 52 steroid-free remission) only mild or severe UC cases were included, and the genes or lncRNAs used were those significantly associated with disease severity in the previous mild vs. severe UC DEseq2 analysis. The randomForest package out-of-box estimate of error rate was used. The outcome analysis was repeated 100 times to get a spread of AUC values. Average mean decrease Gini scores over 100 iterations are shown to priorities severity genes. R AUC(14) package version 0.3.0 was used to calculate the Receiver operating characteristic (ROC) curve and Area under the ROC Curve

(AUC) values. Analysis was performed separately for lncRNAs and protein-coding genes, or for both lncRNAs and proteins coding genes together.

**Identifying co-expressed gene modules**

Weighted gene co-expression network analysis (WGCNA) was implemented to identify modules of co-expressed genes(15, 16), using R WGCNA package version 1.69-81. WGCNA identifies co-expressed gene clusters using pairwise correlations between gene expression profiles. A signed version of WGCNA was used to distinguish between positively and negatively correlated genes. Gene co-expression similarities are converted to signed adjacency values using the power adjacency function, with a β parameter of 5 for lncRNA WGCNA analysis, and a β parameter of 12 for lncRNA and protein-coding genes analysis. The topological similarities represent the interconnectedness of two genes in a given gene co-expression network. Average linkage hierarchical clustering is implemented on TOM-based dissimilarities to detect modules of strongly correlated genes across all samples. The cluster sensitivity parameter (deepSplit) was set to its default value of 2 to identify balanced genes modules while the minimum number of genes in a module (minModuleSize) is set to 30 genes. maxBlockSize is set to 20000 to include all genes in one block. For each module, the first principal component referred to as the eigengene, is considered to be the module representative. A module summarizes the expression levels of all the genes in a given module. Candidate modules are identified based on the strength and significance (Student's asymptotic p-value) of the respective module eigengenes with the phenotypic traits including the disease status. We focused on modules significantly associated with disease status, with $p < 0.001$. The module membership (MM) score signifies the importance of a gene (connectivity-based) within the module and is calculated as the Pearson correlation coefficient between a gene's expression profile and the module eigengene. This analysis was performed separately for lncRNA only, and for lncRNA and protein coding genes. ToppGene(17) and ToppCluster software were used to perform Gene Set Enrichment Analyses (GSEA) of the protein coding genes within the modules in the lncRNA and protein coding genes WGCNA analysis, and visualization of the networks was obtained using Cytoscape.v3.0.2(2).

**PROTECT Microbiome 16S rRNA analysis and association with genes**

16S rRNA reads were processed in a data curation pipeline implemented in QIIME 2 version 2021.4(18, 19). Reads were demultiplexed according to sample specific barcodes. Quality control was performed by truncating reads after three consecutive Phred scores lower than 20. Reads with ambiguous base calls or shorter than 150 bp after quality truncation were discarded. Amplicon sequence variants (ASVs)

detection was performed using Deblur(20), resulting in 156 samples with median of 28,504 reads/sampe (IQR 18,980-43,470). ASV taxonomic classification was assigned using a naive Bayes fitted classifier, trained on the August 2013 Greengenes database for 99% identity 150 bp long reads(21). Severity associated ASVs: differentially abundant ASVs (between mild and severe UC patients) were identified using a paired feature-wise non-parametric rank mean test as implemented in Calour(22) with dsFDR multiple hypothesis correction (FDR < 0.1)(23). For each feature (bacteria), the relative abundance across all samples is ranked. The p-value is calculated by comparing the mean of the ranks for the bacteria in each group to random permutations of the group labels, that are performed only within samples of similar pairing field values. Finally, dsFDR multiple hypothesis correction is applied for the p-values resulting from all the features. Heatmap was generated using Calour with default parameters. Identifying association between lncRNA and microbial ASV: "Hierarchical All-against-All significance testing" (HAllA) version 0.8.20 was used to identify potential correlations between lncRNAs and ASVs associated with disease severity, using Spearman's correlation. 59 mild and severe UC associated ASVs, and the top 30 mild and severe UC associated lncRNAs or protein coding genes, prioritized by DEseq2 FDR were used as input for the HAllA pipeline, with an FDR of 0.1.

**Cell culture and organoids.** Crypts isolation and organoids culturing: L-WRN medium (L-WRN media) was generated using the ATCC mouse fibroblasts cells (L-WRN cells - CRL-3276™), that produce Wnt-3A, R-spondin 3, and noggin(24). To maintain proliferation, TGFBIR inhibitor (SB431542) is added. To initiate differentiation, EP4 inhibitor (L-161,982) is added to DMEM-F12 (Gibco 12634010) medium without FBS. Intestinal biopsies from patients undergoing evaluation via colonoscopy according to approval by the Ethics Committees of the Sheba Medical center were used, after written informed consent were obtained from patient and/or families. Two biopsies were used for crypts isolation. Biopsies were washed 3 times with PBS and Gentamicin/Amphotericin (X 500- corning), cut to small pieces and incubated in cold for 30 minutes with Gentle Cell dissociation Reagent (STEM CELL 100-0485) that helps to release the crypts. Isolated crypts were seeded in Matrigel (Corning 354234) and L-WRN medium to generate the organoids. Cells were maintained at 37°C in a humidified atmosphere containing 5% $CO_2$ and were passaged weekly.

HT29 human colon carcinoma cell lines were purchased from the American Type Culture Collection (Manassas, VA, USA) and maintained in standard culture conditions in DMEM (GIBCO 41965-039, Scotland) containing 10% heat-inactivated fetal bovine serum (GIBCO 12657-029, Scotland). Cells were maintained at 37°C in a humidified atmosphere containing 5% $CO_2$. The following drugs were used

alone and in combination for 24hr to mimic complex disease environment; LPS (100ng/ml(25)), IFNγ (40ng/ml), DFO (100uM), IL1b (25ng/ml), and H2O2 (200uM) for 1 hr.

RNA-seq on HT-29 cells with/without the inflammatory triggers mentioned above, was performed using Lexogen QuantSeq 3' mRNA-Seq libraries sequencing (GSE216810). Principal Coordinates Analysis (PCA) was performed to summarize variation in gene expression between patients. We included 11,489 protein-coding mRNA genes with Reads per Milion (RPM) above 3 in 20% of the samples in our downstream analysis. Differentially expressed genes were determined in GeneSpring® software with fold change differences (FC) ≥1.5 and using the Benjamini–Hochberg false discovery rate correction (FDR≤ 0.05).

qPCR primers that were used for validation:

GAPDH: For: 5'- TGGACCTCATGGCCCACA-3', Rev: 5'- TCAAGGGGTCTACATGGCAA-3'; GATA6-AS1: For: 5'- TTCTGGGAGTCGCGCATT-3', Rev: 5'- GTGGCCGCATTTGGAAAA-3'; HNF1A-AS1: For: 5'- GGAAGCACTTTGACCTCTGC-3', Rev: 5'- AGTGAGCTCTCTCGGTCAGC-3'; CDKN2B-AS1: For: 5'-CTGCTACTGCATAAGTTAGGAAATTG-3', Rev: 5'-CATGTTTACCTTTCTTGTGACTTTTT-3'; LUCAT1: For: 5'-GAGCGAAACTCTGTAGCTCAGCAT-3’, Rev: 5'-TCCTCACAAGAAGCTCACCCAG-3’; LINC01272: For: 5'-GATCCTAGAAAGTGGCAAGGC-3’, Rev: 5'-GTAGGATGTGACTGTGCTGCGTGAC-3’.

**Mouse Models**

C57BL/6, Villin-Cre mice were crossed with HNF1A-AS1 LoxP C57BL/6 mice, which were kindly provided by Prof. Jorge Ferrer's lab(26) from The Barcelona Institute of Science, to generate *HNF1A-AS1*$^{\text{intestine -/-}}$ (intestine-specific deletion of *HNF1A-AS1* promotor in 2 alleles) and *HNF1A-AS1*$^{\text{intestine +/-}}$ (intestine-specific deletion of *HNF1A-AS1* promotor in one of the alleles) animals. Male WT C57BL/6 mice, 8–9 weeks old (cat # 2BL/606, 2BL/606), were purchased from Envigo Laboratories (Jerusalem, Israel). Animals were housed at the Sheba Medical Center animal facility, in a specific pathogen-free environment and fed standard pellet diet and tap water. All procedures performed were in accordance with the Sheba Medical Center’s Guidelines for Animal Studies and approved by the Institutional Animal Ethics Committee (ethical approval code: 006_b16947). To induce colitis, mice were administered drinking water supplemented with dextran sulfate sodium (molecular weight, 36,000–50,000; MP Biomedicals, CAT NO:160110)) for 5 days and were then allowed to recover by drinking unsupplemented water for the next 6 days. Two independent experiment preformed more than 2 months apart are jointly presented. The first experiment included: 5 *HNF1A-AS1*$^{\text{intestine +/+}}$, 3 *HNF1A-AS1*$^{\text{intestine}}$

$^{+/-}$ and 7 *HNF1A-AS1*$^{intestine\ -/-}$ mice who were introduced to DSS. Additionally, this experiment included a control group which wasn't introduced to DSS: 5 *HNF1A-AS1*$^{intestine\ +/+}$, 4 *HNF1A-AS1*$^{intestine\ +/-}$ and 7 *HNF1A-AS1*$^{intestine\ -/-}$ mice. The second experiment included: 10 *HNF1A-AS1*$^{intestine\ +/+}$, 3 *HNF1A-AS1*$^{intestine\ +/-}$ and 10 *HNF1A-AS1*$^{intestine\ -/-}$ mice who were introduced to DSS. The mice were kept in several cages as indicated in the figure and legends, and we also specified the experiment, the litter, and the mother's identity.

The mice were monitored for rectal bleeding. Stool samples were collected at day 1, 6 and 10 for 16S amplicon sequencing. Bleeding score included the fecal occult blood test and visible rectal bleeding, and was graded as 0 = negative,1= weak color (positive by Hemoccult), 2 = strong color (positive by Hemoccult), and 3 = visible rectal blood. At necropsy, the intestines were cut out and measured to determine colon length and weight. 0.5 cm of the rectum were taken for histology and another 0.5 cm of rectum were used for RNA analysis. Tissue sections from the distal colon were fixed in 4% buffered formaldehyde. Paraffin-embedded sections were stained with hematoxylin and eosin. Histopathology scoring was performed by a pathologist in a blinded fashion(27) and graded according to the following parameters: inflammation (0–3; none, slight, moderate, and severe), Extent (0–3; none, mucosa, mucosa and submucosa, and transmural), regeneration (0-4; complete regeneration of normal tissue, almost complete regeneration, regeneration with crypt depletion, surface epithelium not intact, no tissue repair), crypt damage (0–4; none, basal 1/3 damaged, basal 2/3 damaged, only surface epithelium intact, and entire crypt and epithelium lost) and percent involvement (1-4; 1-25%, 26-50%, 51-75%, 76-100%). The score of each parameter was multiplied by the percentage of tissue percent involvement. The final scores of each parameter were added to a sum that was defined as the histology score. The maximum possible histology score was 10.

<u>16S rRNA gene amplicon sequencing and analyses</u>. DNA extraction and PCR amplification of the variable region 4 (V4) of the 16S rRNA gene using Illumina adapted universal primers 515F/806R was conducted using the direct PCR protocol [Extract-N-Amp Plant PCR kit (Sigma-Aldrich, Inc.)](28-30). PCRs were conducted and amplicons were pooled in equimolar concentrations into a composite sample that was size selected (300–500 bp) using agarose gel to reduce non-specific products from host DNA. Sequencing was performed on the Illumina MiSeq platform with the addition of 15% PhiX, and generating paired-end reads of 175b in length in each direction.16S rRNA reads were processed in a data curation pipeline implemented in QIIME 2 version 2021.4(18). Reads were demultiplexed according to sample specific barcodes. Quality control was performed by truncating reads after three consecutive Phred scores lower than 20. Reads with ambiguous base calls or shorter than 150 bp after quality truncation were discarded. Amplicon sequence variants (ASVs) detection was performed using

Deblur(20) with default parameters, resulting in 142 samples with a median of 16799 reads/sample (IQR 9875- 22937). ASV taxonomic classification was assigned using a naive Bayes fitted classifier, trained on the August 2013 Greengenes database for 99% identity on 150 bp long reads(21). Unweighted UniFrac was used as a measure of between sample β-diversity(31), and Faith's phylogenetic diversity(32) was used as a measure of within sample α diversity. using a phylogenetic tree generated by SATé-enabled phylogenetic placement (SEPP)(33). All samples were rarefied to 2000 reads for α and β diversity analysis, to avoid read number effects. The resulting distance matrix was used to perform a principal coordinates analysis (PCoA). Differentially abundant ASVs (between DSS and control or between *HNF1A-AS1*$^{\text{intestine -/-}}$ and *HNF1A-AS1*$^{+/+}$) were identified using a feature-wise non-parametric rank mean test as implemented in Calour(22) with dsFDR multiple hypothesis correction(23) ($FDR \leq 0.1$). For each feature (bacteria), the relative abundance across all samples is ranked. The p-value is calculated by comparing the mean of the ranks for the bacteria in each group to random permutations of the sample labels.

**Summary of statistical tests used.** Statistics used for transcriptomics, microbiome, and metabolomics were performed in R, and details are under these specific sections. Overall, Spearman's rank correlation was used for continuous variables and Mann-Whitney U test for categorical variables, with Benjamini-Hochberg Procedure for FDR correction.

**Study Approval.** We used already published datasets from PROTECT, SEEM, RISK and previously published celiac cohorts. SOURCE was approved by the Sheba Medical center Institutional Review Boards. Informed consent was obtained for all participants.

**Data availability.**

All RNASeq data are deposited in GEO; PROTECT (GSE109142), SOURCE (GSE199906), SEEM (GSE159495), RISK (rectal GSE117993, ileal GSE101794), and celiac cohort (PRJNA528755(9)). An interactive platform of lncRNA expression in celiac disease, Crohn's disease, and ulcerative colitis, and along the gastrointestinal tract [proximal small intestine (duodenum), distal small intestine (ileum), and the large intestine (rectum)] in controls can be found through the R Shiny web interface at https://tzipi.shinyapps.io/lncRNA_gut/.

The mice 16S amplicon sequencing dataset was deposited at the National Center for Biotechnology Information as BioProject PRJNA930578.

Codes are available on github at the following link: https://github.com/Tzipisb/lncRNA_gut_disease

## References


1. Elmentaite R, Ross ADB, Roberts K, James KR, Ortmann D, Gomes T, et al. Single-Cell Sequencing of Developing Human Gut Reveals Transcriptional Links to Childhood Crohn's Disease. *Dev Cell.* 2020;55(6):771-83 e5.
2. Shannon P, Markiel A, Ozier O, Baliga NS, Wang JT, Ramage D, et al. Cytoscape: a software environment for integrated models of biomolecular interaction networks. *Genome Res.* 2003;13(11):2498-504.
3. Smillie CS, Biton M, Ordovas-Montanes J, Sullivan KM, Burgin G, Graham DB, et al. Intra- and Inter-cellular Rewiring of the Human Colon during Ulcerative Colitis. *Cell.* 2019;178(3):714-30 e22.
4. Hyams JS, Davis Thomas S, Gotman N, Haberman Y, Karns R, Schirmer M, et al. Clinical and biological predictors of response to standardised paediatric colitis therapy (PROTECT): a multicentre inception cohort study. *Lancet.* 2019;393(10182):1708-20.
5. Haberman Y, Karns R, Dexheimer PJ, Schirmer M, Somekh J, Jurickova I, et al. Ulcerative colitis mucosal transcriptomes reveal mitochondriopathy and personalized mechanisms underlying disease severity and treatment response. *Nat Commun.* 2019;10(1):38.
6. Haberman Y, Iqbal NT, Ghandikota S, Mallawaarachchi I, Tzipi B, Dexheimer PJ, et al. Mucosal Genomics Implicate Lymphocyte Activation and Lipid Metabolism in Refractory Environmental Enteric Dysfunction. *Gastroenterology.* 2021;160(6):2055-71 e0.
7. Scanlon SA, and Murray JA. Update on celiac disease - etiology, differential diagnosis, drug targets, and management advances. *Clin Exp Gastroenterol.* 2011;4:297-311.
8. Haberman Y, Tickle TL, Dexheimer PJ, Kim MO, Tang D, Karns R, et al. Pediatric Crohn disease patients exhibit specific ileal transcriptome and microbiome signature. *J Clin Invest.* 2014;124(8):3617-33.
9. Leonard MM, Bai Y, Serena G, Nickerson KP, Camhi S, Sturgeon C, et al. RNA sequencing of intestinal mucosa reveals novel pathways functionally linked to celiac disease pathogenesis. *PLoS One.* 2019;14(4):e0215132.
10. Bray NL, Pimentel H, Melsted P, and Pachter L. Near-optimal probabilistic RNA-seq quantification. *Nat Biotechnol.* 2016;34(5):525-7.
11. Soneson C, Love MI, and Robinson MD. Differential analyses for RNA-seq: transcript-level estimates improve gene-level inferences. *F1000Res.* 2015;4:1521.
12. Love MI, Huber W, and Anders S. Moderated estimation of fold change and dispersion for RNA-seq data with DESeq2. *Genome Biol.* 2014;15(12):550.
13. Svetnik V, Liaw A, Tong C, Culberson JC, Sheridan RP, and Feuston BP. Random forest: a classification and regression tool for compound classification and QSAR modeling. *J Chem Inf Comput Sci.* 2003;43(6):1947-58.
14. Michel Ballings DVdP. AUC: Threshold independent performance measures for probabilistic classifiers. 2013.
15. Langfelder P, and Horvath S. WGCNA: an R package for weighted correlation network analysis. *BMC Bioinformatics.* 2008;9:559.
16. Langfelder P, and Horvath S. Fast R Functions for Robust Correlations and Hierarchical Clustering. *J Stat Softw.* 2012;46(11).
17. Chen J, Bardes EE, Aronow BJ, and Jegga AG. ToppGene Suite for gene list enrichment analysis and candidate gene prioritization. *Nucleic Acids Res.* 2009;37(Web Server issue):W305-11.
18. Caporaso JG, Kuczynski J, Stombaugh J, Bittinger K, Bushman FD, Costello EK, et al. QIIME allows analysis of high-throughput community sequencing data. *Nat Methods.* 2010;7(5):335-6.

19. Bolyen E, Rideout JR, Dillon MR, Bokulich N, Abnet CC, Al-Ghalith GA, et al. Reproducible, interactive, scalable and extensible microbiome data science using QIIME 2. *Nature Biotechnology.* 2019;37(8):852-7.
20. Amir A, McDonald D, Navas-Molina JA, Kopylova E, Morton JT, Zech Xu Z, et al. Deblur Rapidly Resolves Single-Nucleotide Community Sequence Patterns. *mSystems.* 2017;2(2).
21. Bokulich NA, Kaehler BD, Rideout JR, Dillon M, Bolyen E, Knight R, et al. Optimizing taxonomic classification of marker-gene amplicon sequences with QIIME 2's q2-feature-classifier plugin. *Microbiome.* 2018;6(1):90.
22. Xu ZZ, Amir A, Sanders J, Zhu Q, Morton JT, Bletz MC, et al. Calour: an Interactive, Microbe-Centric Analysis Tool. *mSystems.* 2019;4(1).
23. Jiang L, Amir A, Morton JT, Heller R, Arias-Castro E, and Knight R. Discrete False-Discovery Rate Improves Identification of Differentially Abundant Microbes. *mSystems.* 2017;2(6).
24. VanDussen KL, Sonnek NM, and Stappenbeck TS. L-WRN conditioned medium for gastrointestinal epithelial stem cell culture shows replicable batch-to-batch activity levels across multiple research teams. *Stem Cell Res.* 2019;37:101430.
25. Guo S, Al-Sadi R, Said HM, and Ma TY. Lipopolysaccharide causes an increase in intestinal tight junction permeability in vitro and in vivo by inducing enterocyte membrane expression and localization of TLR-4 and CD14. *Am J Pathol.* 2013;182(2):375-87.
26. Beucher A, Miguel-Escalada I, Balboa D, De Vas MG, Maestro MA, Garcia-Hurtado J, et al. The HASTER lncRNA promoter is a cis-acting transcriptional stabilizer of HNF1A. *Nat Cell Biol.* 2022;24(10):1528-40.
27. Dieleman LA, Palmen MJHJ, Akol H, Bloemena E, Pena AS, Meuwissen SGM, et al. Chronic experimental colitis induced by dextran sulphate sodium (DSS) is characterized by Th1 and Th2 cytokines. *Clin Exp Immunol.* 1998;114(3):385-91.
28. Braun T, Di Segni A, BenShoshan M, Neuman S, Levhar N, Bubis M, et al. Individualized Dynamics in the Gut Microbiota Precede Crohn's Disease Flares. *Am J Gastroenterol.* 2019.
29. Caporaso JG, Lauber CL, Walters WA, Berg-Lyons D, Huntley J, Fierer N, et al. Ultra-high-throughput microbial community analysis on the Illumina HiSeq and MiSeq platforms. *ISME J.* 2012;6(8):1621-4.
30. Braun T, Di Segni A, BenShoshan M, Asaf R, Squires JE, Farage Barhom S, et al. Fecal microbial characterization of hospitalized patients with suspected infectious diarrhea shows significant dysbiosis. *Sci Rep.* 2017;7(1):1088.
31. Lozupone C, and Knight R. UniFrac: a new phylogenetic method for comparing microbial communities. *Appl Environ Microbiol.* 2005;71(12):8228-35.
32. Faith DP. Systematics and Conservation: On Predicting the Feature Diversity of Subsets of Taxa. *Cladistics.* 1992;8(4):361-73.
33. Janssen S, McDonald D, Gonzalez A, Navas-Molina JA, Jiang L, Xu ZZ, et al. Phylogenetic Placement of Exact Amplicon Sequences Improves Associations with Clinical Information. *mSystems.* 2018;3(3).
